# Seasonal influenza vaccination in older people: A systematic review and meta-analysis of the determining factors

**DOI:** 10.1371/journal.pone.0234702

**Published:** 2020-06-18

**Authors:** George N. Okoli, Otto L. T. Lam, Florentin Racovitan, Viraj K. Reddy, Christiaan H. Righolt, Christine Neilson, Ayman Chit, Edward Thommes, Ahmed M. Abou-Setta, Salaheddin M. Mahmud

**Affiliations:** 1 George & Fay Yee Centre for Healthcare Innovation, University of Manitoba, Winnipeg, Manitoba, Canada; 2 College of Pharmacy, Rady Faculty of Health Sciences, University of Manitoba, Winnipeg, Manitoba, Canada; 3 Vaccine and Drug Evaluation Centre, University of Manitoba, Winnipeg, Manitoba, Canada; 4 Community Health Sciences, Max Rady College of Medicine, Rady Faculty of Health Sciences, University of Manitoba, Winnipeg, Manitoba, Canada; 5 Neil John Maclean Health Sciences Library, University of Manitoba, Winnipeg, Manitoba, Canada; 6 Sanofi Pasteur, Swiftwater, Pennsylvania, United States of America; 7 Leslie Dan Faculty of Pharmacy, University of Toronto, Toronto, Ontario, Canada; 8 Department of Mathematics and Statistics, University of Guelph, Guelph, Ontario, Canada; Leibniz Institute for Prevention Research and Epidemiology BIPS, GERMANY

## Abstract

**Background/Objectives:**

Despite influenza vaccination programs in various jurisdictions, seasonal influenza vaccine (SIV) uptake remains suboptimal among older people (≥65years old), an important subpopulation for influenza vaccination. We sought to summarize determinants of SIV uptake (any vaccine receipt) and vaccination adherence (receipt of vaccine in two or more seasons in sequence) among older people.

**Methods:**

We searched for population-based studies conducted in community-dwelling older people (irrespective of their health status) from 2000–2019. Two reviewers independently selected publications for inclusion. One reviewer extracted data from the included studies; a second checked the extracted data for errors. Disagreements were resolved by discussion and consensus, or a third reviewer. We were interested in the determinants of SIV uptake and vaccination adherence. Where appropriate, we pooled adjusted results using the inverse variance, random-effects method and reported the odds ratios (OR) and their 95% confidence intervals (CI).

**Results:**

Out of 11,570 citations screened, we included 34 cross-sectional studies. The following were associated with increased SIV uptake: being older (OR 1.52, 95%CI 1.38–1.67 [21 studies]), white (1.30, 1.14–1.49 [10 studies]), married (1.23, 1.17–1.28 [9 studies]), non-smoker (1.28, 1.11–1.47 [7 studies]), of a higher social class (1.20, 1.06–1.36 [2 studies]), having a higher education (1.12, 1.04–1.21 [14 studies]), having a higher household income (1.11, 1.05–1.18 [8 studies]), having a chronic illness (1.53, 1.44–1.63 [16 studies]), having poor self-assessed health (1.23, 1.02–1.40 [9 studies]), having a family doctor (2.94, 1.79–4.76 [2 studies]), and having health insurance (1.58, 1.13–2.21 [6 studies]). The influence of these factors varied across geographical regions. Being older (1.26, 1.11–1.44 [2 studies]) was also associated with increased vaccination adherence.

**Conclusions:**

Several factors may determine SIV uptake and vaccination adherence among older people. More studies are needed to provide a stronger evidence base for planning more effective influenza vaccination programs.

## Introduction

Influenza remains a major public health issue, with substantial disease and economic burden. The World Health Organization (WHO) estimates that there are three to five million cases of severe seasonal influenza illness with about 290,000 to 650,000 deaths globally every year. [1] Most of the deaths occur among older people (≥65 years old). [2]

To reduce the impact of seasonal influenza outbreaks, many countries have implemented annual vaccination programs. [3] Seasonal influenza vaccination is highly recommended for groups most at risk of influenza complications, including older people, pregnant women, the very young, and people with chronic diseases. [4, 5] In many countries, influenza vaccination is also highly recommended for health care workers to reduce transmission between workers and patients, and especially to protect older patients with higher risk of complications. Some countries have introduced free annual vaccination for some of the recommended population subgroups, while others have introduced universal vaccination programs which offer free annual vaccination to all individuals six months old and above. Despite these programs, seasonal influenza vaccine (SIV) uptake among older people remains suboptimal even in jurisdictions with universal influenza vaccination programs. In North America for example, only about 65% of older people in Canada received SIV during the 2015/16 [6] and 2016/17 seasons, [7] despite a national vaccination coverage goal of 80%, [8] and a WHO vaccination coverage goal of 75% for this subpopulation. [9] The proportion of older people vaccinated annually against influenza in Canada is consistent with estimates of vaccination among this subpopulation in the United States of America (USA) [10, 11] and elsewhere. [11] The number of those who consistently receive SIV annually is likely lower, although not known with certainty. [12]

Probable determinants of SIV uptake and vaccination adherence among older people have been suggested, but the available evidence has not been well summarized. In addition to an individual’s knowledge, perceptions and attitudes towards influenza vaccination, [13, 14] some socio-demographic characteristics, as well as health systems and primary care provider characteristics, could either facilitate or hinder vaccine uptake and adherence. Understanding the influence of these characteristics among older people is essential for designing effective interventions to improve vaccination in this important population subgroup. [15] It is also necessary for public health decision-making to support effective planning, optimization and evaluation of influenza vaccination programs.

We aimed to systematically appraise and summarize findings of published studies on determinants of SIV uptake and vaccination adherence among older people.

## Methods

We undertook a systematic review and meta-analysis following the Cochrane Handbook for Systematic Reviews of Interventions guidelines, [16] and reported our findings following the Preferred Reporting Items for Systematic reviews and Meta-Analyses (PRISMA) guidelines. [17] We registered our systematic review in the international prospective register of systematic reviews (PROSPERO) prior to execution of the review search strategy (CRD42018086803). Our review methods have been reported in a previous publication on the determinants of SIV uptake among older people in the United States. [15] This present review is an update to the previous review, using an updated search and broader inclusion criteria in order to include evidence from any country (as compared to only the USA in the previous publication [n = 5 studies]).

### Study eligibility criteria

We considered only studies conducted from 2000–2019 and which recruited community-based older people, irrespective of their health status. Our decision to search for studies conducted from 2000 onwards was because a publicly funded influenza vaccination program was first introduced in the late 1990s; for example, in 1999 in Canada, [18] and from 1993 in the USA. [19] We considered only full-text publications in English that reported on socio-demographic and/or health-related factors influencing SIV uptake (any vaccine receipt) or vaccination adherence (receipt of vaccine in two or more seasons in sequence). Non-community-based studies, such as studies conducted in personal care homes, hospitals, prisons, etc., were excluded.

### Search strategy and study selection

A knowledge synthesis librarian (CN) designed a search strategy for MEDLINE (Ovid). This was peer-reviewed by a second, independent librarian using the PRESS checklist. [20] The revised search strategy for MEDLINE (S1 Table) was then adapted by the knowledge synthesis librarian for Embase (Ovid), CINAHL (EbscoHost) and Scopus (Elsevier) bibliographic databases. Relevant websites such as the USA Centers for Disease Control and Prevention, British Columbia Centre for Disease Control, WHO-International, Canadian Sentinel Practitioner Surveillance Network, and the Network of European Influenza Monitoring Vaccine Effectiveness (I-MOVE) were also searched for articles. We initially searched for literature in January 2018. We then updated the searches on the 7^th^ of January, 2020. Identified citations from the searches were managed in EndNote software version X9. The citations were independently screened for eligibility by two reviewers in pairs (GNO/FR; OLTL/VKR) using a two-stage sifting approach to review the titles/abstracts and full-text articles. The screening was conducted in a specially designed Microsoft (MS) Access 2016 database (Microsoft Corporation, Redmond, WA, USA). The number of ineligible citations at the abstract screening stage and both the number and reason for ineligibility at the full-text article screening stage were recorded. Disagreements were resolved by discussion and consensus or a third reviewer (GNO/AMAS).

### Data extraction

Data extraction was carried out in MS Excel 2016 (Microsoft Corporation, Redmond, WA, USA) and was first piloted on a small selection of studies (n = 6) by two reviewers in pairs (GNO/FR; OLTL/VKR) prior to commencing full extraction. One reviewer extracted data from the included studies and a second reviewer independently checked the extracted data for errors (GNO/FR/OLTL/VKR). Disagreements were resolved by discussion and consensus or a third reviewer (GNO/AMAS). The extracted data included study details (first author, year of study, year of publication, country, region, funding source), population information (size, average age and sex), outcome (factors associated with SIV uptake/vaccination adherence, and their statistical measures), results (multivariable adjusted effect estimates and associated 95% confidence intervals [CI]) and details relevant to study quality assessment.

### Study quality assessment

Two reviewers in pairs (GNO/FR; OLTL/VKR) independently assessed study quality using the National Institutes of Health (NIH) quality assessment tool for observational cohort and cross-sectional studies. [21] Disagreements were resolved by discussion and consensus, or a third reviewer (GNO/AMAS). The NIH quality assessment tool assesses 14 criteria to determine study quality, including clarity of the research question and objectives; appropriateness of the study population and participants’ selection; sample size justification; and quality of measurements and data analysis. A study was judged to be of high quality if it satisfied all assessed parameters; of good quality if it satisfied all but one parameter; of moderate quality if it did not satisfy two to four parameters; and of poor quality if it did not satisfy more than four parameters.

### Data synthesis and analysis

Data synthesis and analysis were conducted by one reviewer (GNO) and reviewed for errors by a data analyst (CHR). We summarized relevant study characteristics and quality assessments in a tabular form. Where appropriate, we pooled data using the inverse variance, random-effects model implemented in STATA (version 13; StataCorp LP, Texas, USA) and reported the pooled adjusted odds ratios (OR) and their 95% confidence intervals (CI). Adjusted relative risks, as well as adjusted rate and prevalence ratios (reported by only 3 studies) [22–24], were pooled with adjusted ORs. Statistical heterogeneity between pooled results was assessed and quantified using the I-squared statistic (I^2^). [25] Where appropriate, publication bias was assessed visually using funnel plots and, statistically, using Egger’s regression test. [26] We reported on publication bias only if detected. Where pooling was not possible, data were synthesized narratively. For uniformity in the pooled analysis, where necessary, we flipped an OR (1/OR) and the associated CI (1/upper CI and 1/lower CI) for a reverse comparison. For example, if a majority of the studies compared females against males, and a study compared males against females, the result from the study was flipped to represent a comparison of females against males, in line with the majority. If a study reported results for more than one comparison of the same determinant, for example, the 70–79 and 80–89 years age groups compared with the 65–69 years age group, results from the two comparisons were first pooled using a fixed-effects model before being pooled with other results for comparison of older against younger age groups using a random-effects model.

## Results

**Fig 1:**
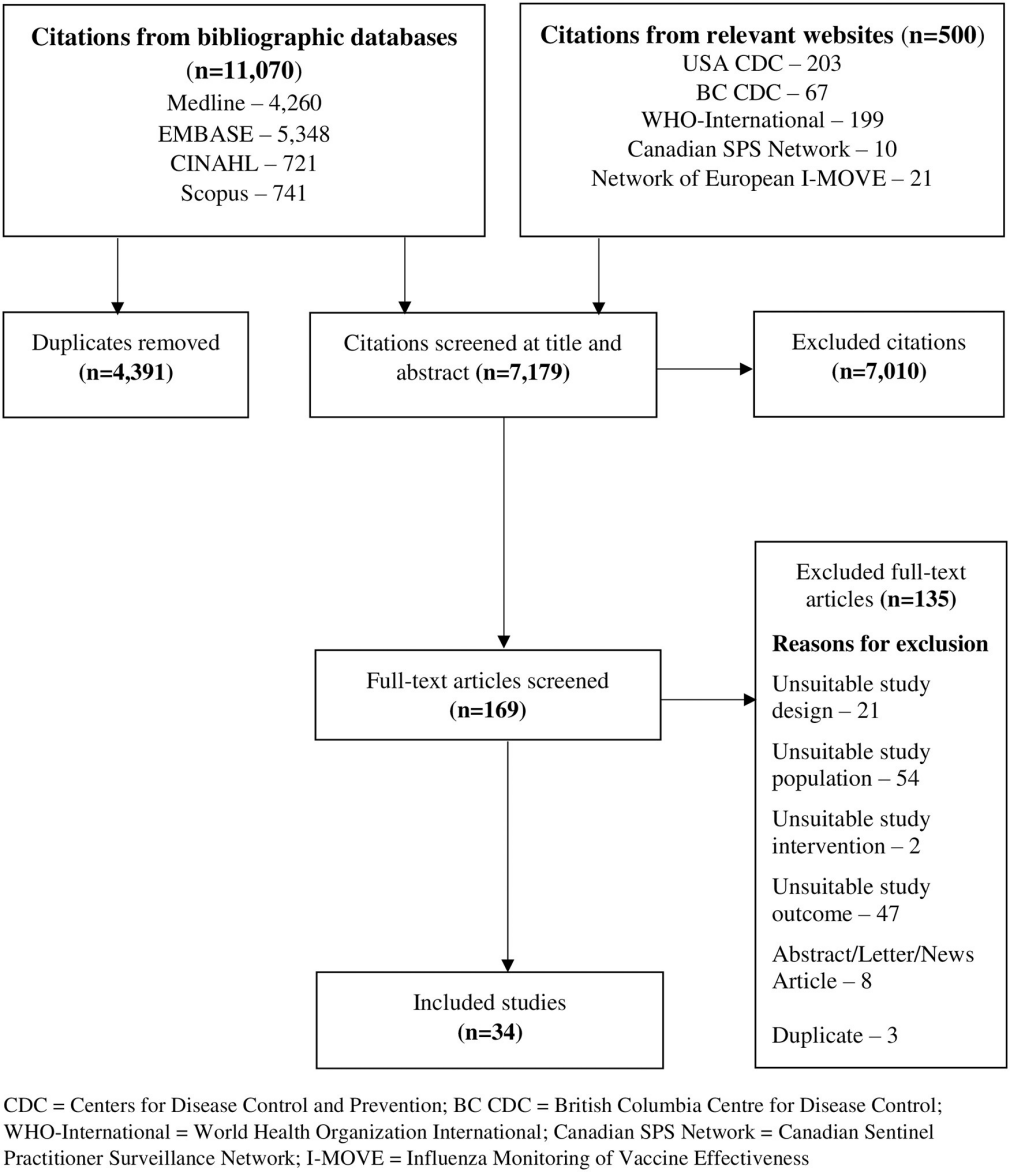
Summary of literature search and screening process (PRISMA flow diagram). CDC = Centers for Disease Control and Prevention; BC CDC = British Columbia Centre for Disease Control; WHO-International = World Health Organization International; Canadian SPS Network = Canadian Sentinel Practitioner Surveillance Network; I-MOVE = Influenza Monitoring of Vaccine Effectiveness.

Out of a total of 9,990 retrieved citations, 34 cross-sectional studies met our eligibility criteria (Fig 1). [22–24, 27–57] Table 1 is a summary of the characteristics of these studies. Six studies were conducted in Spain, [27, 33, 35, 40, 41, 48] seven in the USA, [28, 31, 39, 47, 49, 50, 55] and four in Hong Kong. [32, 36, 44, 45] There were two studies each from Italy [24, 37] and South Korea, [46, 52] and one study each from the United Kingdom (UK), [29] Thailand, [23] Canada, [51] Israel, [34] Taiwan, [42] France, [43] Australia, [22] Japan, [57] Switzerland, [53] Singapore, [54] Serbia, [56] Europe (involving 11 countries) [30] and Ireland (both the Republic of Ireland and Northern Ireland). [38] These studies were conducted from 2000 to 2018, and published from 2004 to 2019. Three studies examined only determinants of seasonal influenza vaccination adherence, [40, 42, 43] one study examined determinants of both SIV uptake and vaccination adherence, [41] and the rest of the studies examined only determinants of SIV uptake. One study involved only female participants, [27] while the rest of the studies involved both sexes. Study population size ranged from 354 to 13,106,163 individuals and this information was not reported in one study. [49] Overall, the studies examined individual socio-demographic characteristics, clinical factors, health behaviours and organizational determinants of SIV uptake and/or vaccination adherence, focusing mainly on receipt of influenza vaccination in the previous year for assessment of SIV uptake. All but one study (not included in the pooled analyses) [43] utilized multivariable data analysis and adjusted for varied socio-demographic and health-related covariates. One study each reported relative risk, [22] rate ratio, [24] and prevalence ratio, [23] while the rest of the studies reported ORs. One study was funded by industry, [49] while 20 studies were funded by non-industry sources. [22, 23, 28, 30, 33, 35, 36, 38, 40–43, 45–47, 50, 51, 54, 56, 57] Five studies reported no funding, [24, 34, 37, 52, 53] and eight studies did not disclose funding information. [27, 29, 31, 32, 39, 44, 48, 55] Data from five studies from the USA were summarized in our previous publication. [15]

**Table 1:** Characteristics of included studies (N = 34).

Study	Year Country (Region)	Funding	Sample size (% Male)	Adjusted covariates (Data source)	Factors assessed
**Pena-Rey 2004** [27]	2000 Spain (Galicia)	NR	1,111 (0%)	Age, population size of place of residence, income, marital status, self-assessed health status, visit to physician, tetanus vaccination, having a caregiver (Women’s Social and Health survey)	Age; population size of place of residence; income; marital status; self-assessed health status; visit to physician; having a caregiver
**Chen 2005** [28]	2000–2001 USA (California)	Robert Wood Johnson Foundation	10,724 (NR)	Race/ethnicity, health insurance type, healthcare access, health status, health risk behavior, gender, marital status, education, annual household income, place of birth (Health Interview survey)	Race/ethnicity
**Burns 2005** [29]	2001–2002 UK (Birmingham)	NR	444 (45%)	Age, sex, living arrangements, household occupational status, chronic disease, smoking, alcohol consumption (Structured interview)	Age; sex; living with others; household occupational status; chronic disease; smoking; alcohol consumption
**Landi 2005** [30]	2001–2003 Europe (11 European countries)	European Union	3,878 (26%)	Age, sex, living alone, economic problem, compromised activities of daily living, impaired cognitive performance, depression, malnutrition, chronic disease (Multi-linked database)	Age; sex; marital status; living alone; economic problem; impaired cognitive performance; chronic disease
**Appel 2006** [31]	2001–2003 USA (Denver)	NR	740 (35%)	Gender, primary insurance, medical and psychiatric comorbidities, primary care site, age at end of audit year, primary care visit frequency (Chart audits)	Age; chronic disease; health insurance; hospital visits
**Lau 2006** [32]	2004 China (Hong Kong)	NR	877 (45%)	Not specified (Telephone survey using structured questionnaire)	Age; education
**de Andres 2007** [33]	2003 Spain (National)	Carlos III Institute of Public Health Spain	6,134 (64.4%)	Not specified (National health survey)	Age; sex; size of town or city; perceived health status; chronic disease; smoking
**Abramson 2008** [34]	2006–2007 Israel (Jerusalem)	Not funded	29,447 (43%)	Age, sex, chronic disease, marital status, Jewish practices, complementary health insurance, patient’s physician, influenza vaccination status, age, sex, specialty, place of graduation/specialiSation (Computerized health databases)	Age; sex; chronic disease; marital status; complimentary health insurance
**Jimenez-Garcia 2008** [35]	2004–2005 Spain (Madrid)	Carlos III Institute of Public Health Spain	1,629 (NR)	Not specified (Health survey)	Age; sex; nationality; chronic disease
**Lau 2008** [36]	2004 China (Hong Kong)	Chinese University of Hong Kong	483 (43.7%)	Socio-demographic factors, health status, living arrangements, perception related to intravenous injection, and others not specified (longitudinal survey using structured questionnaire)	Age; sex; level of education; living with family members; living with spouse; perceived health status; visited clinic; chronic disease; independent living
**Chiatti 2011** [37]	2004–2005 Italy (National)	Not funded	24,760 (43.1%)	Sex, age, education, marital status, household wealth, smoking, chronic diseases, health status, respondent type, help from (family, friends, neighbours), household size, private domestic worker (home helper) (Health survey using a self-administered questionnaire)	Age; sex; smoking; chronic disease; self-reported health status; household size; marital status
**Crawford 2011** [38]	2010 Republic of Ireland & Northern Ireland (National)	Irish Health Research Board	2,033 (43.4%)	Age, marital status, and others not reported (Multidisciplinary healthy research program data)	Age; sex; marital status; living status; social class; residential location
**Takayama 2012** [39]	2009 USA (National)	NR	134,101 (NR)	Age, sex, race, marital status, education, annual income, health care coverage, smoking, physical activity, fruit and vegetable consumption, body mass index, frequency of poor physical health, history of hypertension, arthritis, asthma, diabetes, coronary heart disease, myocardial infarction, or stroke (Surveillance system database)	Age; race/ethnicity; marital status; level of education; annual income; healthcare coverage (insurance); smoking status; chronic disease
**Martinez-Baz 2012** [40]	2009–2011 Spain (Navarre)	ECDC and the Spanish Ministry of Health	64,245 (44%)	Age, sex, residence, country of origin, outpatient visits in the previous 12 months, chronic conditions, chronic condition in the previous 12 months, hospitalisation in the previous 12 months; dependence, living with children < 15 years (Computerized medical records)	Age; sex; residence location; immigration status; outpatient visits in previous 12 months; chronic disease; level of dependency; living with children
**Aguilar 2012** [41]	2010–2011 Spain (Navarre)	ECDC and the Carlos III Institute of Public Health Spain	104,427 (43.8%)	Age, sex, major chronic conditions, visits as outpatient in the previous year, immigrant status, place of residence, level of dependency, hospitalisation in the previous year (Computerised medical records)	Age; sex; major chronic disease; visit outpatient in the previous year; immigration status; residence; independency; 2009/10 seasonal influenza vaccination
**Chang 2013** [42]	2005–2007 Taiwan, Republic of China (National)	National Science Council, Taiwan, Republic of China	1,384 (54.5%)	Age, sex, educational level, marital status, living arrangement, employment, household monthly income, urbanization; social support, daily-living difficulty level, self-assessed health, number of chronic diseases, out-patient visits during the preceding year, hospitalisation during the preceding year, medical seeking behavior, smoking, drinking, exercise during the preceding 2 weeks, use of preventive health examination during the preceding year (National health interview survey)	Age; sex; education; marital status; living arrangement; employment; household income; residence location; chronic disease; outpatient visits during preceding year; smoking; drinking range; self-assessed health
**Caille-Brillet 2014** [43]	2009–2011 France (National)	Varied French government departments	269 (47.7%)	Receipt of seasonal influenza vaccine (SIV) (CoPanFlu France cohort)	Vaccination with 2007–2008 SIV; vaccination with 2008–2009 SIV; vaccination with 2009–2010 SIV
**Chan 2015** [44]	2011–2012 China (Hong Kong)	NR	4,204 (47.1%)	Sex, economic status, education level, smoking habit, health status, chronic illnesses, history of medical consultation, subscription to health insurance and subsidy, household characteristics (Population-based survey data)	Sex; economic status; level of education; chronic disease; medical consultation in past year; smoking; living alone; household size; having medical insurance; housing
**Dyda 2015** [22]	2012 Australia (New South Wales)	NHMRC	12,314 (59.6%)	Age, sex, country of birth, place of residence, level of education, annual household income, carer status, smoking, body mass index, medical indication for influenza vaccination (Australian universal health insurance database)	Age; sex; residence location; level of education; annual household income; carer; smoking; bmi; independence; siv indication
**Mo 2015** [45]	Not clear but after 2009 China (Hong Kong)	Department of Health, Hong Kong	1,101 (38.7%)	Not specified (Telephone survey)	Sex; chronic disease
**Kwon 2016** [46]	2007–2009 South Korea (National)	KNHANES IV	3,567 (40.7%)	Not specified (Nationwide National Health and Nutrition Examination Survey database)	Age; level of education; household income; living alone; smoking; drinking; self-reported health status; limitation in daily activities
**Dios-Guerra 2017** [48]	2009/2011/2014 Spain (National)	NR	18,442 (NR)	Not specified (National health survey)	Sex; social class; level of education
**Henning-Smith 2017** [50]	2012 USA (273 Counties)	US Department of Health and Human Services	6,027 (NR)	Community and individual characteristics (Medicare Current Beneficiary Survey and County Health Rankings data)	Residence location
**Praphasiri 2017** [23]	2014 Thailand (Nakhon Phanom Province)	Centers for Disease Control and Prevention	581 (40%)	Functional status and health belief model variables (Provincial population-based survey data)	Age; education; marital status
**Khan 2018** [47]	2011–2015 USA (Florida)	NCBDDDH	13,106,163 (50.4%)	Age, gender, education; employment, income, marital status, medicare, regular doctor, year of survey (behavioural risk factor surveillance system database)	Age; sex; race/ethnicity; level of education; employment status; annual income; marital status; hospital visits; disability
**La 2018** [49]	2011–2014 USA (10 States)	GlaxoSmithKline Biologicals SA	Unclear (NR)	State of residence, socio-demographic status, health status characteristics, health behaviours (Behavioural risk factor surveillance system database)	Age; sex; level of education; health status; last checkup
**Roy 2018** [51]	2013–2014 Canada (National)	Statistics Canada	33,664 (NR)	Age, education, household income, country of birth, aboriginal identity, mother tongue, area of residence, having family doctor, has a chronic medical condition, self-perceived health (Canadian Community Health Survey (CCHS))	Age; education; household income; chronic disease; residence location; health status; having a family physician
**Seo 2018** [52]	2005–2014 South Korea (National)	Not funded	10,590 (NR)	Sex, area of residency, level of education, house income, age, number of diseases (Korea National Health and Nutrition Examination Surveys [KNHANES])	Sex; education; household income; residence location
**Fabiani 2019** [24]	2016–2017 Italy (Lazio)	Not funded	1,255,657 (NR)	Reference general practitioner, vaccination time-interval, and others not clearly reported (The Lazio region Regional Health Authority registry and other linked health databases)	Age; sex
**Zurcher 2019** [53]	2007–2012 Switzerland (National)	Not funded	Unclear (NR)	Age group, sex, body mass index, language region, setting, citizenship, education level, smoking, self-reported health status, hospital stay insurance, use of any alternative medicine, chronic diseases, healthcare profession (The Swiss Health Interview Survey [SHIS])	Age; sex; education; smoking status; chronic disease; bmi; nationality; residential location; health status
**Ho 2019** [54]	2017–2018 Singapore (National)	National Medical Research Council, Ministry of Health, Singapore	8,837 (45%)	Age, gender, ethnicity, housing type, follow-up for various chronic diseases, and others not clearly defined (Data from a pragmatic, cluster-randomized crossover trial)	Age; sex; chronic disease
**Lu 2019** [55]	2010–2016 USA (National)	NR	Unclear (NR)	Age groups, education, U.S.-born status, and other factors not clearly reported (The National Health Interview Survey)	Ethnicity
**Gazibara 2019** [56]	2012–2013 Serbia (Belgrade)	Ministry of Education and Science, Republic of Serbia	354 (44%)	Years of schooling, household monthly income, place or residence, medications used, and flu knowledge score (Data from a community health Center-recruited individuals)	Marital status
**Chinzorig 2019** [57]	2017 Japan (National)	Ministry of Health, Labour and Welfare, Japan	474 (52%)	Sex, self-reported health status, marital status and income (A web-based survey)	Sex; marital status; education; household income; health status

NR = Not Reported; ECDC = European Centre for Disease Prevention and Control; NHMRC = National Health and Medical Research Council; KNHANES IV = The Korean National Health and Nutrition Examination Survey IV; NCBDDDH = National Center on Birth Defects and Developmental Disabilities

### Study quality assessment

Table 2 is a summary of quality assessments of the included studies. Four studies were judged to be of high quality having satisfied all assessed parameters. [23, 28, 54, 56] Twenty-one studies did not report on one assessed parameter (sample size justification) and were therefore judged to be of good quality. [22, 27, 29–31, 34, 36–38, 40–44, 47, 50–53, 55, 57] Seven studies did not report on sample size justification and did not provide a list of the confounders that were measured and adjusted for in their analyses, and were judged to be of moderate quality. [24, 32, 33, 35, 45, 46, 48] A further two studies did not report on sample size justification and also did not apply participant eligibility uniformly, and were also judged to be of moderate quality. [39, 49]

**Table 2:** Study quality assessment.

Study	Research objective stated	Study population specified	Participation rate ≥50%	Eligibility uniformly applied	Sample size justification	Exposures measured prior to outcomes	Sufficient study timeframe	Measured different levels of exposure	Consistent exposure measure across participants	Exposure assessed more than once over time	Outcome measures clearly defined	Blinding of outcome assessors	≤20% Loss to follow-up	Confounders measured and adjusted
Pena-Rey 2004 [27]	✔	✔	NA	✔	×	✔	NA	✔	✔	NA	✔	NA	NA	✔
Chen 2005 [28]	✔	✔	NA	✔	✔	✔	NA	✔	✔	NA	✔	NA	NA	✔
Burns 2005 [29]	✔	✔	NA	✔	×	✔	NA	✔	✔	NA	✔	NA	NA	✔
Landi 2005 [30]	✔	✔	NA	✔	×	✔	NA	✔	✔	NA	✔	NA	NA	✔
Appel 2006 [31]	✔	✔	NA	✔	×	✔	NA	✔	✔	NA	✔	NA	NA	✔
Lau 2006 [32]	✔	✔	NA	✔	×	✔	NA	✔	✔	NA	✔	NA	NA	NR
de Andres 2007 [33]	✔	✔	NA	✔	×	✔	NA	✔	✔	NA	✔	NA	NA	NR
Abramson 2008 [34]	✔	✔	NA	✔	×	✔	NA	✔	✔	NA	✔	NA	NA	✔
Jimenez-Garcia 2008 [35]	✔	✔	NA	✔	×	✔	NA	✔	✔	NA	✔	NA	NA	NR
Lau 2008 [36]	✔	✔	NA	✔	×	✔	NA	✔	✔	NA	✔	NA	NA	✔
Chiatti 2011 [37]	✔	✔	NA	✔	×	✔	NA	✔	✔	NA	✔	NA	NA	✔
Crawford 2011 [38]	✔	✔	NA	✔	×	✔	NA	✔	✔	NA	✔	NA	NA	✔
Takayama 2012 [39]	✔	✔	NA	×	×	✔	NA	✔	✔	NA	✔	NA	NA	✔
Martinez-Baz 2012 [40]	✔	✔	NA	✔	×	✔	NA	✔	✔	NA	✔	NA	NA	✔
Aguilar 2012 [41]	✔	✔	NA	✔	×	✔	NA	✔	✔	NA	✔	NA	NA	✔
Chang 2013 [42]	✔	✔	NA	✔	×	✔	NA	✔	✔	NA	✔	NA	NA	✔
Caille-Brillet 2014 [43]	✔	✔	NA	✔	×	✔	NA	✔	✔	NA	✔	NA	NA	✔
Chan 2015 [44]	✔	✔	NA	✔	×	✔	NA	✔	✔	NA	✔	NA	NA	✔
Dyda 2015 [22]	✔	✔	NA	✔	×	✔	NA	✔	✔	NA	✔	NA	NA	✔
Mo 2015 [45]	✔	✔	NA	✔	×	✔	NA	✔	✔	NA	✔	NA	NA	NR
Kwon 2016 [46]	✔	✔	NA	✔	×	✔	NA	✔	✔	NA	✔	NA	NA	NR
Dios-Guerra 2017 [48]	✔	✔	NA	✔	×	✔	NA	✔	✔	NA	✔	NA	NA	NR
Henning-Smith 2017 [50]	✔	✔	NA	✔	×	✔	NA	✔	✔	NA	✔	NA	NA	✔
Praphasiri 2017 [23]	✔	✔	NA	✔	✔	✔	NA	✔	✔	NA	✔	NA	NA	✔
Khan 2018 [47]	✔	✔	NA	✔	×	✔	NA	✔	✔	NA	✔	NA	NA	✔
La 2018 [49]	✔	✔	NA	×	×	✔	NA	✔	✔	NA	✔	NA	NA	✔
Roy 2018 [51]	✔	✔	NA	✔	×	✔	NA	✔	✔	NA	✔	NA	NA	✔
Seo 2018 [52]	✔	✔	NA	✔	×	✔	NA	✔	✔	NA	✔	NA	NA	✔
Fabiani 2019 [24]	✔	✔	NA	✔	×	✔	NA	✔	✔	NA	✔	NA	NA	NR
Zurcher 2019 [53]	✔	✔	NA	✔	×	✔	NA	✔	✔	NA	✔	NA	NA	✔
Ho 2019 [54]	✔	✔	NA	✔	✔	✔	NA	✔	✔	NA	✔	NA	NA	✔
Lu 2019 [55]	✔	✔	NA	✔	×	✔	NA	✔	✔	NA	✔	NA	NA	✔
Gazibara 2019 [56]	✔	✔	NA	✔	✔	✔	NA	✔	✔	NA	✔	NA	NA	✔
Chinzorig 2019 [57]	✔	✔	NA	✔	×	✔	NA	✔	✔	NA	✔	NA	NA	✔

NA = not applicable; NR = not reported (although confounders were adjusted, a clear list of the confounders was not reported)

### Individual socio-demographic determinants of SIV uptake

**Fig 2:**
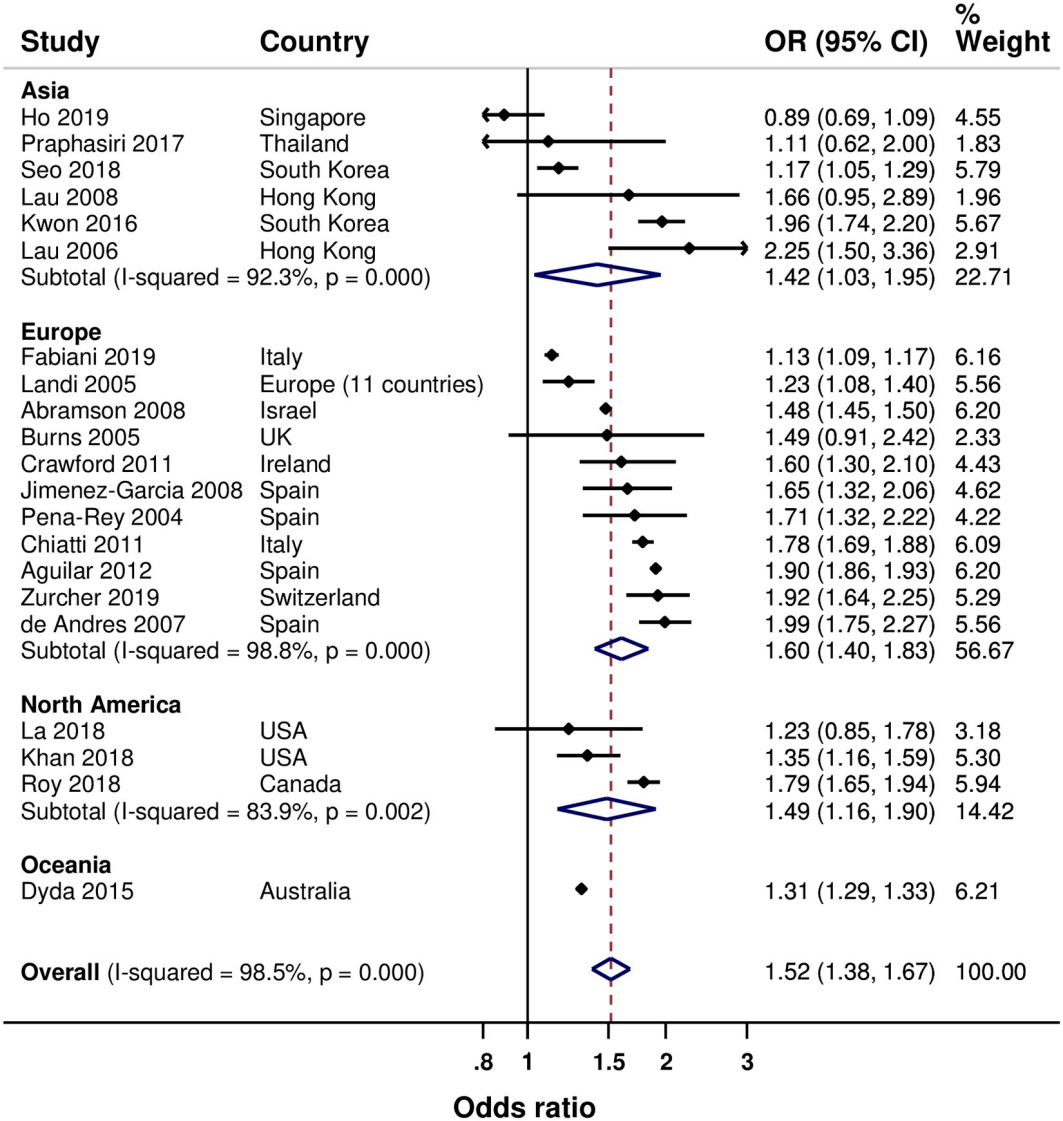
Forest plot of the association between age and SIV uptake (older versus younger).

Results of the meta-analyses are summarised in Table 3. Twenty-one studies examined age. [22–24, 27, 29, 30, 32–38, 41, 46, 47, 49, 51–54] Overall, older age (in groups) was associated with increased SIV uptake by 52% (95% CI: 38% to 67%), with high heterogeneity between study results (Fig 2). Age-associated increased SIV uptake was found in Asia (by 42%), Europe (by 60%) and North America (by 49%), with also high heterogeneity between the study results, although most studies in each of the regions reported statistically significant increased SIV uptake.

**Table 3:** Results from meta-analysis of association between reported determinants and SIV uptake.

Determinants	Comparison	Continent	Number of studies	Population size	Pooled Odds Ratio (95% CI)	I^2^ Statistic (%)
Age	Older vs. Younger	Asia	6	24,935	**1.42 (1.03–1.95)**	92.3
		Europe	11	Over 1.5 million	**1.60 (1.40–1.83)**	98.8
		North America	3	Over 13.1 million	**1.49 (1.16–1.90)**	83.9
		Oceania	1	12,314	1.31 (1.29–1.33)*	NA
Ethnicity	Non-Whites vs. Whites	North America	1	134,101	**0.88 (0.85–0.91)**	84.7
	African Americans vs. Whites	North America	5	Unclear	**0.69 (0.51–0.93)**	87.1
	Asians vs. Whites	North America	2	Unclear	1.01 (0.50–2.05)	80.6
	Hispanics vs. Whites	North America	2	Unclear	**0.69 (0.53–0.89)**	63.6
Marital status	Married vs. Not married	Asia	2	1,055	1.59 (0.91–2.78)	0
		Europe	5	57,705	**1.23 (1.16–1.30)**	14.5
		North America	2	13,240,264	1.10 (0.89–1.37)	0
Education	Higher vs. Lower	Asia	7	20,776	1.23 (0.98–1.56)	76.8
		Europe	2	Over 18,442	1.10 (0.67–1.80)	91.3
		North America	4	Over 13.2 million	**1.22 (1.02–1.47)**	83.1
		Oceania	1	12,314	1.02 (0.99–1.05)*	NA
Household income	Low vs. High	Asia	3	14,631	**0.91 (0.84–0.99)**	0
		Europe	1	1,111	0.72 (0.53–0.98)*	NA
		North America	3	13,273,928	**0.85 (0.76–0.96)**	87.7
		Oceania	1	12,314	0.99 (0.92–1.02)*	NA
Sex	Female vs. Male	Asia	6	25,689	**1.23 (1.05–1.44)**	66.4
		Europe	11	Over 1.4 million	**0.93 (0.91–0.97)**	60.2
		North America	2	Over 13.1 million	1.03 (0.94–1.14)	0
		Oceania	1	12,314	1.04 (1.02–1.07)*	NA
Living situation	Living vs. Not living alone	Asia	3	8,254	0.96 (0.69–1.35)	78.1
		Europe	3	6,355	**0.70 (0.51–0.96)**	68.9
Residence	Small vs. Large population area	Asia	1	10,590	1.19 (1.08–1.32)*	NA
		Europe	5	Over 113,705	0.94 (0.79–1.11)	83.9
		North America	2	39,691	**0.77 (0.69–0.87)**	11.9
		Oceania	1	12,314	0.99 (0.97–1.01)*	43.6
Social class	High vs. Low	Europe	2	20,475	**1.20 (1.06–1.36)**	23.5
Immigration	Immigrants vs. Natives	Europe	3	Over 106,056	0.69 (0.34–1.40)	93.6
Comorbidity status	Having vs. Not having a chronic disease	Asia	5	25,215	**1.65 (1.32–2.07)**	82.9
		Europe	8	Over 170,719	**1.49 (1.38–1.60)**	83.1
		North America	3	34,404	**1.50 (1.15–1.94)**	57.2
Cognitive performance	Impaired vs. Not impaired	Asia	2	4,050	1.18 (0.99–1.41)	0
		Europe	2	4,989	**0.68 (0.59–0.78)**	0
		Oceania	1	12,314	1.05 (1.02–1.09)*	NA
Smoking	Smokers vs. Non-smokers	Asia	2	7,771	**0.64 (0.42–0.97)**	80.2
		Europe	3	Over 30,894	0.76 (0.58–1.00)	86.2
		North America	1	134,101	0.94 (0.89–0.99)*	NA
		Oceania	1	12,314	0.78 (0.62–0.98)*	NA
Alcohol consumption	Regular vs. Never or not regular	Asia	1	3,567	0.86 (0.72–1.04)*	NA
		Europe	1	444	1.48 (0.85–2.57)*	NA
Self-assessed health	Poor vs. Good health	Asia	3	4,524	1.08 (0.66–1.77)	69.7
		Europe	4	Over 32,005	**1.32 (1.07–1.63)**	78.3
		North America	2	Over 33,664	0.63 (0.10–3.95)	92.8
Health insurance	Having vs. Not having health insurance	Asia	1	4,204	1.05 (0.85–1.29)*	NA
		Europe	1	29,447	2.23 (2.12–2.36)*	NA
		North America	4	13,285,379	**1.40 (1.25–1.55)**	0.1
Healthcare provider	Have not vs. Have a regular physician	North America	2	13,139,827	**0.34 (0.21–0.56)**	77.8

* = odds ratio (not pooled odds ratio); NA = not applicable; Unclear = data not reported for one study

**Fig 3:**
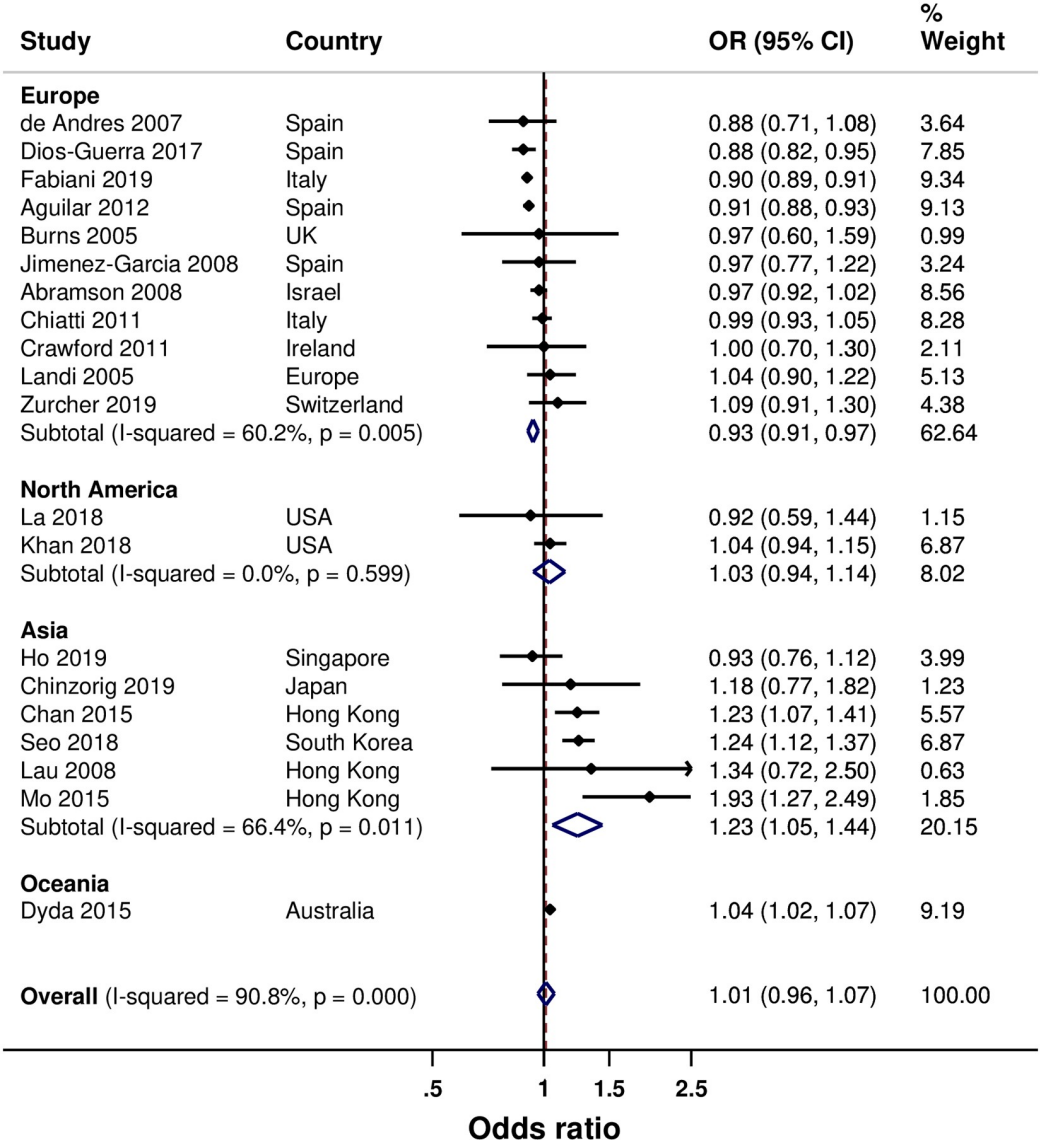
Forest plot of the association between sex and SIV uptake (female versus male).

Twenty studies examined sex. [22, 24, 29, 30, 33–38, 41, 44, 45, 47–49, 52–54, 57] Overall, being female was not significantly associated with increased SIV uptake (Fig 3) but there was evidence of publication bias (p = 0.027). However, being female in Asia was associated with a 23% increased SIV uptake, whereas a 7% decreased SIV uptake was found in Europe (with evidence of publication bias; p = 0.036). A non-significant increased association was found in North America. Six studies (all from North America) examined the influence of ethnicity. [28, 31, 39, 47, 49, 55] Compared with being white, being non-white was associated with a 23% decrease in SIV uptake. The associated decrease in SIV uptake was higher for being an African American or Hispanic (both by 31%); however, with substantial heterogeneity between the pooled results (S1 Fig). A non-significant increase in SIV uptake was found for being Asian. However, one of the two studies that contributed to the pooled analysis reported decreased SIV uptake and the other reported increased SIV uptake for being Asian. Nine studies examined influence of marital status. [23, 27, 34, 37–39, 47, 56, 57] Overall, being married was associated with an increased SIV uptake by 23% (17% to 28%); a 23% increase in Europe, and a non-significant increase in Asia and North America (S2 Fig).

The influence of education was examined by 14 studies. [22, 23, 32, 36, 39, 44, 46–49, 51–53, 57] Overall, having a higher education was associated with a modest increased SIV uptake by 12% (4% to 21% [with evidence of publication bias; p = 0.038]), a 22% increase in North America (with high heterogeneity), and a non-significant increased SIV uptake in Asia and Europe (S3 Fig).

Eight studies examined the influence of household income. [22, 27, 39, 46, 47, 51, 52, 57] Overall, a low household income was associated with a small but substantial 10% decrease in SIV uptake (5% to 15%). A similar association was found in Asia (decrease by 9%) and North America (decrease by 15%) (S4 Fig). Six studies examined the influence of living situation. Compared with not living alone, living alone was associated with a 30% decrease in SIV uptake in Europe (S5 Fig). A non-significant decreased SIV uptake was found in Asia.

Nine studies examined the influence of area of residence. [22, 27, 33, 38, 41, 50–53] Overall, compared with living in an urban area, living in a rural or low populated area was associated with a non-significant decrease in SIV uptake (S6 Fig). The same observation was made in Europe, but a 23% decrease was found in North America. Two studies (both from Europe) examined the influence of social class. [38, 48] A high social class was associated with a 20% increase in SIV uptake (S7 Fig). Three studies (all from Europe) examined the influence of immigration status. [35, 41, 53] A non-significant decreased SIV uptake was found for immigrants versus natives (S8 Fig).

### Health-related determinants of SIV uptake

**Fig 4:**
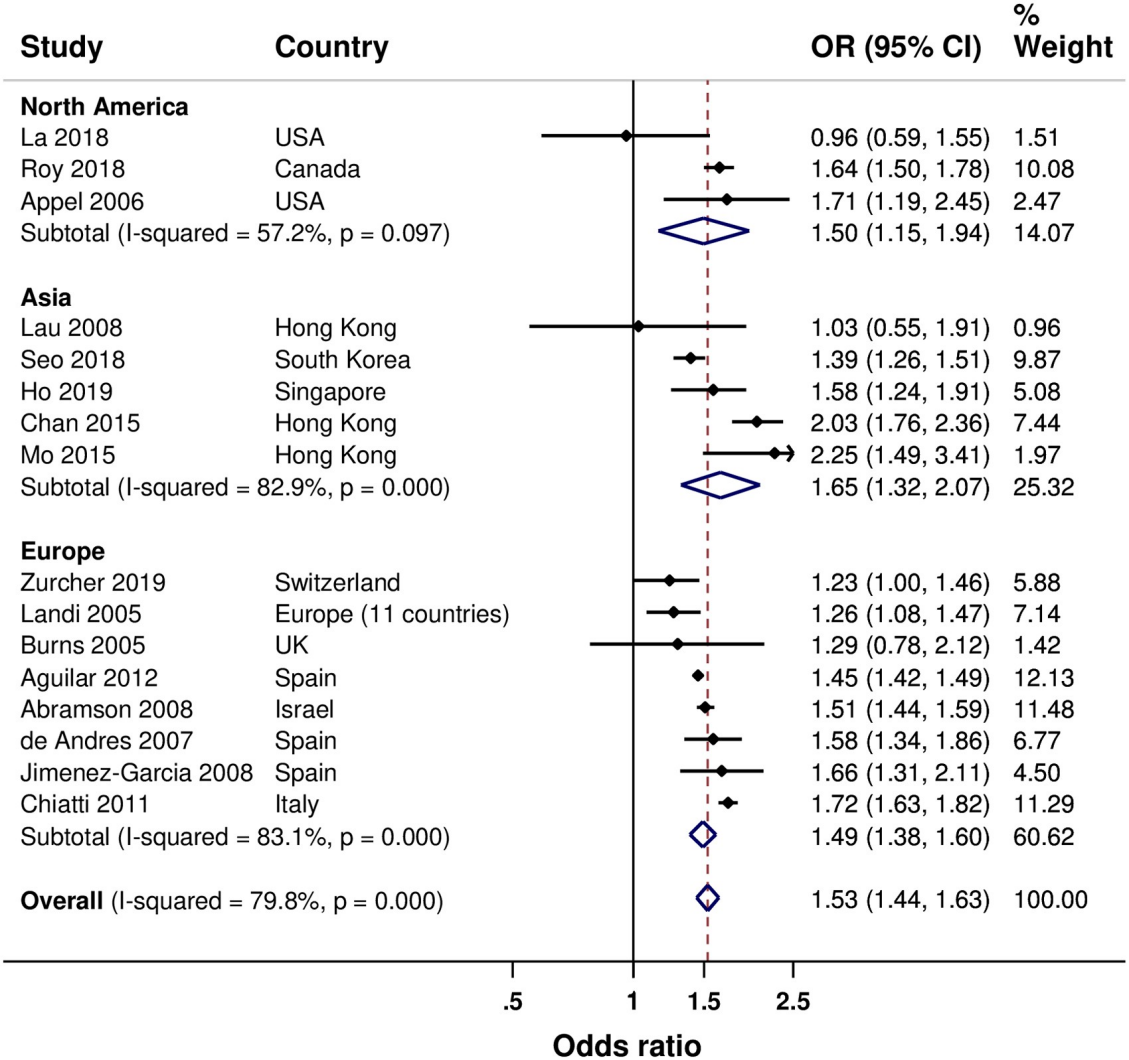
Forest plot of the association between chronic disease and SIV uptake (having versus not having a chronic disease).

Results of the meta-analyses are summarized in Table 3. Sixteen studies examined the influence of having a chronic disease(s). [29–31, 33–37, 41, 44, 45, 49, 51–54] Overall, having a chronic disease(s) was associated with increased SIV uptake by 53% (44% to 63%). A similar association was found in Asia (by 65%), Europe (by 49%), and North America (by 50%); however, with substantial heterogeneity between pooled results for Europe and Asia, although most studies in the regions reported statistically significant increased SIV uptake (Fig 4). Five studies examined the influence of cognitive impairment. [22, 27, 30, 36, 46] Overall, a non-significant decrease in SIV uptake was found for having a cognitive impairment, and the same observation was found in Asia. However, an associated 32% decrease was found in Europe (S9 Fig).

Seven studies examined the influence of smoking status. [22, 33, 37, 39, 44, 46, 53] Generally, smoking was associated with a decreased SIV uptake by 22% (10% to 32%), and a 36% decrease in SIV uptake in Asia (with substantial heterogeneity) (S10 Fig). A non-significant decreased SIV uptake was found in Europe.

Nine studies examined the influence of self-assessed health status. [27, 33, 36, 37, 46, 49, 51, 53, 57] Overall, poor self-assessed health was associated with an increased SIV uptake by 23% (2% to 47%), and a 32% increase in SIV uptake in Europe (S11 Fig). A non-significant increased and decreased SIV uptake was found in Asia and North America, respectively.

Six studies examined the influence of health insurance. [28, 31, 34, 39, 44, 47] Overall, having health insurance was associated with an increased SIV uptake by 58% (13% to 121%); however, with high heterogeneity between the pooled results (S12 Fig). An associated 40% increase was found in North America. Two studies (both from North America) examined the influence of having a healthcare provider (family doctor). [47, 51] Compared with having a family doctor, not having a family doctor was associated with a 66% decrease in SIV uptake (S13 Fig). There was one study each from Asia and Europe for alcohol consumption, and these reported a non-significant decrease and increase in SIV uptake, respectively, for regular compared with irregular or non-alcohol drinkers.

### Determinants of adherence to seasonal influenza vaccination

Table 4 is a summary of the results. Of four studies that examined seasonal influenza vaccination adherence, one study each from Asia and Europe reported data suitable for meta-analysis. [40, 42] Older age was associated with a 26% increase in vaccination adherence. There was insufficient data to fully examine other determinants.

**Table 4:** Results from meta-analysis of association between reported determinants and seasonal influenza vaccination adherence.

Determinant	Comparison made	Continent	Number of studies	Population size	OR/within study POR (95% CI)	I^2^ Statistic (%)
Marital status	Married vs. Not married	Asia	1 (2 results)	1,384	1.02 (0.78–1.32)	0
Education	Higher vs. Lower	Asia	1 (4 results)	1,384	0.80 (0.62–1.03)	0
Household income	High vs. Low	Asia	1 (4 results)	1,384	1.07 (0.89–1.30)	0
Household	Living vs. Not living alone	Asia	1 (2 results)	1,384	0.85 (0.56–1.29)	0
Social class	High vs. Low	Asia	1 (2 results)	1,384	**1.30 (1.01–1.56)**	0
Immigration	Immigrants vs. Natives	Europe	1	64,245	0.55 (0.45–0.67)	–
Employment	Employed vs. Not employed	Asia	1 (2 results)	1,384	0.83 (0.60–1.16)	0
Smoking	Smokers vs. Non-smokers	Asia	1 (2 results)	1,384	1.20 (0.87–1.65)	0
Alcohol consumption	Consumption vs. No consumption	Asia	1 (2 results)	1,384	**1.36 (1.02–1.80)**	0
Self-assessed health	Poor vs. Good health	Asia	1 (2 results)	1,384	1.29 (0.89–1.87)	0
Consultation in past year	Outpatient consultation vs. None	Asia	1 (2 results)	1,384	**2.07 (1.58–2.72)**	0

## Discussion

Our review indicates that age, race/ethnicity, level of education, marital status, social class, household income, smoking status, morbidity, self-assessed health status, health insurance, and primary health care may determine SIV uptake, and that age also may determine seasonal influenza vaccination adherence. Our findings suggest that regional variations in the effects of some of these determinants may exist. However, it is important to note that this review examined only the determinants reported in the included studies; therefore, the findings should not be interpreted as an exhaustive assessment of all potentially relevant factors that could determine SIV uptake and vaccination adherence. Compared with our previous publication on the determinants of SIV uptake among older people in the USA (a commissioned study), [15] this present review is an update using a broader inclusion criteria in order to include evidence from any country (as compared to only the USA in our previous publication [n = 5 studies]). As such, this present review includes 29 additional studies, including two additional studies from the USA. [50, 55]

Our findings that older individuals were more likely to take up and to adhere to seasonal influenza vaccination were similar to the findings by Ryu and colleagues, [58] and may reflect increased incidence of chronic diseases among the very old, which potentially leads to having more frequent contact with healthcare providers and an increased likelihood of being offered SIV. Whereas being female was found to be associated with an increased SIV uptake in Asia, the opposite was found in Europe. However, the studies from Asia were mainly from Hong Kong while those from Europe were mainly from Spain and Italy. Nevertheless, the point estimates of the contributing study results were fairly consistent in each region, with a majority of those from Asia reporting increased SIV uptake, and a majority of those from Europe reporting decreased SIV uptake for females. We could not identify any differing demographic or health system-specific characteristics between the Asian and the European countries that may inform the observed difference in the effect of sex on SIV uptake. Studies have shown that females are more likely than males to visit primary care physicians, [59, 60] although a UK study found no difference in older people. [61] Frequent visits to the physician have been shown to be associated with an increased receipt of SIV. [38] Studies have also suggested a slightly higher morbidity in older females compared with males in some populations, [62, 63] with a study from China suggesting that older females have more comorbidities than males. [64] Since morbidity is a determinant of increased SIV uptake, as reported in this review (as well as other studies), the observed variation in the association of sex with SIV uptake in Asia compared with Europe could be a result of more morbidity among older females compared with males in the studies from Asia.

Overall, living situation made no difference to SIV uptake. However, living alone was found to be associated with a significantly decreased SIV uptake in Europe. This finding supports a large national study that found that living alone is associated with the worst care experience and immunization among older people. [65] Living alone has also been shown to be a predictor of functional decline, and worsening or development of chronic diseases in older people. [66] Our finding that having a chronic disease is associated with increased SIV uptake in Asia, Europe and North America is in line with previous literature. [67, 68] The strong associations found between chronic disease(s) and SIV uptake in these regions may be explained by the fact that, in addition to older people being recommended for seasonal influenza vaccination in all of the jurisdictions in which the contributing studies were conducted, individuals with chronic disease(s) were also a highly recommended subgroup for seasonal influenza vaccination in all of these jurisdictions. Furthermore, it is highly probable that there were seasonal influenza vaccination programs in all of the jurisdictions during the study periods. Although an associated increased SIV uptake was found for higher education overall, including in North America, a non-significant increased SIV uptake was found in Europe and Asia. These findings may be explained by the wide variations between grouping of educational attainment and the reference education attainment groups in the included studies for these regions. It may also be explained by differences in health system characteristics, including availability of free-of-charge seasonal influenza vaccination for older people in the specific jurisdictions.

Studies from North America (all from the USA) showed that, compared with Whites, non-Whites had a lower SIV uptake. Ethnicity correlates with many socioeconomic factors associated with SIV uptake. Studies have found that ethnicity is associated with educational achievement, [69–72] employment status, and income. [73–75] Access and utilization patterns of healthcare services in the USA have also been found to be influenced by ethnicity, [76, 77] income, [78, 79] and education. [80, 81] Furthermore, an individual’s ethnicity, employment status and income have been found to determine the extent of their health insurance coverage. [82] Overall health status has also been linked with ethnicity. [83] All these suggest that ethnicity is an independent determinant of SIV uptake, especially in the USA. However, the definition of ethnicity is highly debatable, and the measure of ethnicity often differs between studies. [84, 85] Therefore, this impacts any conclusions that could be made regarding the influence of ethnicity on SIV.

The determinants of SIV uptake among older people as found in this study are similar to those that have been reported for other vaccines among this subpopulation; for example, human papillomavirus, [86] pneumococcal vaccines, [87, 88] and also for varied vaccines for subgroups such as adult diabetics. [89] In addition, Nagata et al. examined only social determinants of SIV in older people globally, and found that age, sex, marital status, education, ethnicity, socioeconomic status, place of residence, and perceived health status determined SIV uptake. [90] This was also a systematic review but differed significantly from ours in that both qualitative and quantitative studies were included in the review, and the evidence was synthesized narratively. Dyda et al. systematically reviewed factors associated with influenza vaccination among Australian adults and found that being aged ≥65 years old was a determinant of SIV uptake compared with being <65 years old. [67] Similar to our findings, they also found that having a medical risk factor determined SIV uptake. Dyda and colleagues also reported that lower education and lower income were associated with increased SIV uptake. Yeung et al. systematically reviewed factors associated with the uptake of SIV in adults 18–64 years. [68] Although the age group that they studied differed significantly from that of our review, nonetheless, similar to our findings, they found that advancement in age and having chronic diseases were strongly indicative of SIV uptake. Jain et al. systematically appraised and quantified social factors associated with vaccine uptake among Europeans aged ≥60 years. Similarly to our review, they reported a higher uptake of SIV for individuals not living alone; individuals with a higher income and education; natives compared with immigrants; and those living in more affluent neighbourhoods compared with those living in deprived areas. [91] The included studies in our review varied in the way SIV uptake was confirmed. They also varied in the way some of the determinants were assessed. For example, measurement of educational attainment, household income and social class (higher versus lower) varied across studies. SIV uptake and some socio-demographic and health-related factors were confirmed through medical records in some of the studies whereas self-reported in other studies.

Due to the inadequacy of data, we could not determine the factors that independently influenced SIV uptake, although many of the factors examined were likely strongly correlated. While some differences may have existed between health systems and population characteristics of the countries in which the studies were conducted, they were nearly all developed countries with comparable health systems. We could not assess the impact of social desirability reporting and recall biases which are inherent in self-reporting. Within and across regional studies, it was difficult to separate individual effects from regional effects (for example, effectiveness of vaccine policies and programs), since the studies were conducted in different jurisdictions with potentially unique jurisdictional characteristics. There were also differences between studies in the categorization of some of the assessed factors and the reference groups used in some of the analyses. It was not possible to determine the effect of any country-specific characteristics on the the assessed factors. We could also not ascertain with certainty if any of the countries did not have an influenza vaccination program in place before the study. Having or not having a vaccination program in place could influence SIV uptake and vaccination adherence in that awareness and affordability of vaccination would likely be of much influence if the latter was the case. Furthermore, there were differences in how statistical analysis models were constructed in studies, including the covariates that were adjusted for. All of the above may explain the heterogeneity in some of our pooled analyses. A limited number of studies contributed to the assessment of many of the determinants within geographical regions. The paucity of data also meant that we could not substantially examine determinants of vaccination adherence. Considering the above, the generalizability of our findings within and across geographical regions may not be totally appropriate since the studies included in the review were neither fully representative of the countries in which they were conducted, nor the geographical regions to which the countries belong.

A lack of resources to support the professional translation of articles published in languages other than English meant that we limited the inclusion of studies to full-text articles published in English. Considering the global coverage of our review, this was a potential limitation because any non-English publications could have been excluded. The decision to include only publications from the year 2000 onwards may have also limited the number of potentially relevant studies for our review. However, this allowed us to focus on literature published after influenza vaccination became publicly funded and freely available for older people in many Western jurisdictions, which were among the first to introduce publicly funded influenza vaccination programs. Inadequacy of data did not allow us to assess some of the important determinants of SIV uptake and vaccination adherence in some regions. It also did not allow for subgroup analysis by study characteristics as we had originally planned, and the examination of publication bias for many of the assessed determinants. Finally, we reported this review in accordance with the PRISMA guidelines, although the reporting guidelines for Meta-analysis of Observational Studies in Epidemiology (MOOSE) may be more appropriate. [92] However, MOOSE guidelines are currently out-of-date (several decades old to date) and have not been updated to reflect changes in the field.

Notwithstanding the aforementioned limitations, our review has many merits. In full compliance with the Cochrane Handbook for Systematic Reviews of Interventions, a detailed review methods was developed and duly registered on the PROSPERO website prior to the execution of our search strategy. We utilized the expertise of a professional knowledge synthesis librarian in developing a sensitive search strategy for the review and this was subsequently peer-reviewed and by an independent knowledge synthesis librarian using the PRESS checklist. We searched appropriate bibliographic databases for literature, properly sifted retrieved citations, and assessed the quality of the included studies using a validated and appropriate tool. For the countries with more than one publication, we checked for potential overlap in the study populations and confirmed that there were none. Our review provides more insight that could aid both clinicians and public health policy makers in their decision-making to improve older people’s care and access to seasonal influenza vaccination, respectively. Furthermore, the review reveals the lack of research on the determinants of seasonal influenza vaccination adherence among older people.

## Conclusions

The available evidence suggests that many socio-demographic and health-related factors are associated with SIV uptake among older people. The influence of these factors appears to vary between geographical regions. There also appears to be a relationship between age and seasonal influenza vaccination adherence among older people. More studies are necessary for a stronger evidence base to support the planning of more effective influenza vaccination programs for older people.

## Supporting information

S1 Table Search strategy for ovid medline(R) epub ahead of print, in-process & other non-indexed citations, ovid medline(R) daily and ovid medline(R) <1946 to present>. (DOCX)

S1 Fig Forest plot of the association between ethnicity and SIV uptake (ethnic minorities versus whites). (TIF)

S2 Fig Forest plot of the association between marital status and SIV uptake (married versus not married). (TIF)

S3 Fig Forest plot of the association between education and SIV uptake (higher versus lower education). (TIF)

S4 Fig Forest plot of the association between household income and SIV uptake (low versus high income). (TIF)

S5 Fig Forest plot of the association between living situation and SIV uptake (living versus not living alone). (TIF)

S6 Fig Forest plot of the association between residence and SIV uptake (living in small [rural] versus large [urban] population area). (TIF)

S7 Fig Forest plot of the association between social class and SIV uptake (high versus low social class). (TIF)

S8 Fig Forest plot of the association between immigration and SIV uptake (immigrants versus natives). (TIF)

S9 Fig Forest plot of the association between cognitive performance and SIV uptake (impaired versus not impaired). (TIF)

S10 Fig Forest plot of the association between smoking and SIV uptake (smokers versus non-smokers). (TIF)

S11 Fig Forest plot of the association between self-assessed health and SIV uptake (poor versus good self-assessed health). (TIF)

S12 Fig Forest plot of the association between health insurance and SIV uptake (having versus not having health insurance). (TIF)

S13 Fig Forest plot of the association between having a family doctor and SIV uptake (have not versus have a regular family doctor). (TIF)

S1 Checklist PRISMA 2009 checklist. (DOC)
