# Supplementary material for: Seasonal influenza vaccination in older people: A systematic review and meta-analysis of the determining factors
Source: PLoS One. 2020 Jun 18;15(6):e0234702. doi: 10.1371/journal.pone.0234702 (PMC7302695; doi:10.1371/journal.pone.0234702)
Supplement: S1 Table — (DOCX) [file pone.0234702.s001.docx]

### **S1 Table. Search strategy for Ovid MEDLINE(R) epub ahead of print, in-process & other non-indexed citations, Ovid MEDLINE(R) daily and Ovid MEDLINE(R) <1946 to Present>.**

| **#** | **Searches** |
| --- | --- |
| 1 | Influenza Vaccines/ |
| 2 | Influenza, Human/pc |
| 3 | ("flu shot" or "flu shots").ti,ab,kf. |
| 4 | (Antiinfluenza or "anti influenza").ti,ab,kf. |
| 5 | or/1-4 |
| 6 | Influenza, Human/ |
| 7 | exp influenzavirus a/ |
| 8 | exp influenzavirus b/ |
| 9 | flu.ti,ab,kf. |
| 10 | Influenza*.ti,ab,kf. |
| 11 | or/6-10 |
| 12 | Vaccines/ |
| 13 | exp Immunization Programs/ |
| 14 | inoculat*.ti,ab,kf. |
| 15 | vaccin*.ti,ab,kf. |
| 16 | immuni*.ti,ab,kf. |
| 17 | or/12-16 |
| 18 | 5 or (11 and 17) |
| 19 | exp aged/ |
| 20 | Elder*.ti,ab,kf. |
| 21 | Senior*.ti,ab,kf. |
| 22 | aging.ti,ab,kf. |
| 23 | Geriatric*.ti,ab,kf. |
| 24 | (Retired or retiree*).ti,ab,kf. |
| 25 | ("post menopausal" or postmenopausal).ti,ab,kf. |
| 26 | ((Old or older or oldest) adj3 (people or person* or patient* or individual* or adult*)).ti,ab,kf. |
| 27 | "old old".ti,ab,kf. |
| 28 | "oldest old".ti,ab,kf. |
| 29 | ("65" adj3 (age* or years)).ti,ab,kf. |
| 30 | (hexagenarian* or sexagenarian* or septuagenarian* or octogenarian* or nonagenarian* or centenarian* or supercentenarian*).ti,ab,kf. |
| 31 | or/19-30 |
| 32 | "Patient Acceptance of Health Care"/ |
| 33 | patient compliance/ |
| 34 | medication adherence/ |
| 35 | exp Socioeconomic Factors/ |
| 36 | Health Knowledge, Attitudes, Practice/ |
| 37 | (Uptake or "up take").ti,ab,kf. |
| 38 | Adhere*.ti,ab,kf. |
| 39 | (Complian* or Comply or complied).ti,ab,kf. |
| 40 | accept*.ti,ab,kf. |
| 41 | Predict*.ti,ab,kf. |
| 42 | Factor*.ti,ab,kf. |
| 43 | Facilitat*.ti,ab,kf. |
| 44 | Enabl*.ti,ab,kf. |
| 45 | encourag*.ti,ab,kf. |
| 46 | motivat*.ti,ab,kf. |
| 47 | help*.ti,ab,kf. |
| 48 | (succeed or success*).ti,ab,kf. |
| 49 | Determinant*.ti,ab,kf. |
| 50 | characteristic*.ti,ab,kf. |
| 51 | indicator*.ti,ab,kf. |
| 52 | (demographic* or sociodemographic*).ti,ab,kf. |
| 53 | ((improv* or increas* or rise or rais* or optimal* or optimi* or higher or influenc*) adj4 (level* or coverage or rate* or access*)).ti,ab,kf. |
| 54 | ((improv* or increas* or rise or rais* or optimal* or optimi* or higher or influenc*) adj4 (probability or chance*)).ti,ab,kf. |
| 55 | (barrier* or obstacle* or hinder* or hesitan* or hesitat* or refus* or noncomplian* or "non complian*" or deter* or discourag* or challeng*).ti,ab,kf. |
| 56 | or/32-55 |
| 57 | 18 and 31 and 56 |
| 58 | limit 57 to english language |
| 59 | limit 58 to yr="2000 - Current" |
| 60 | remove duplicates from 59 |
| 61 | 60 not (exp animals/ not humans/) |
