# Supplementary figures and images for: Seasonal influenza vaccination in older people: A systematic review and meta-analysis of the determining factors
Source: PLoS One. 2020 Jun 18;15(6):e0234702. doi: 10.1371/journal.pone.0234702 (PMC7302695; doi:10.1371/journal.pone.0234702)

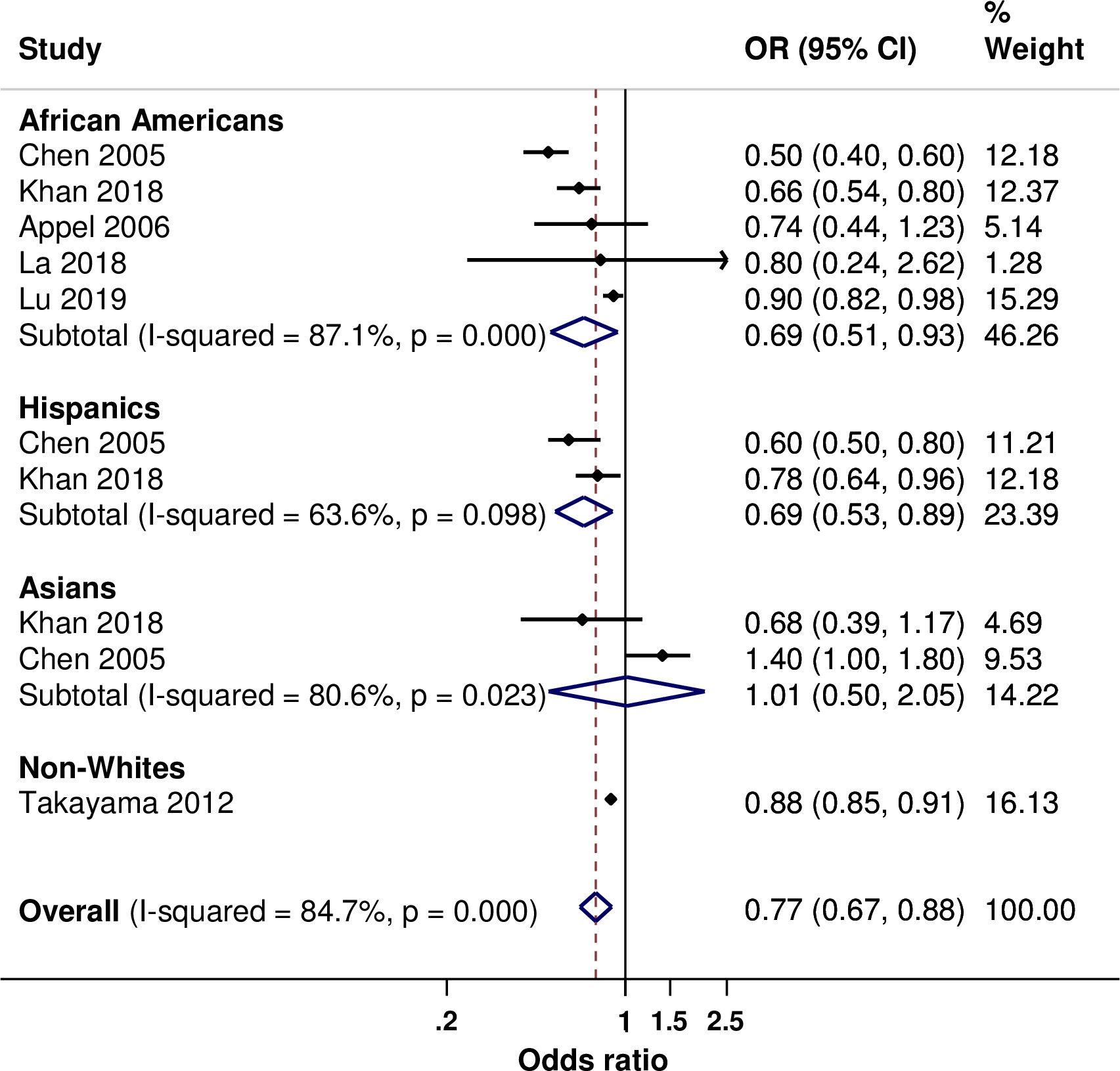

Supplement: S1 Fig — (TIF) [file pone.0234702.s002.tif]

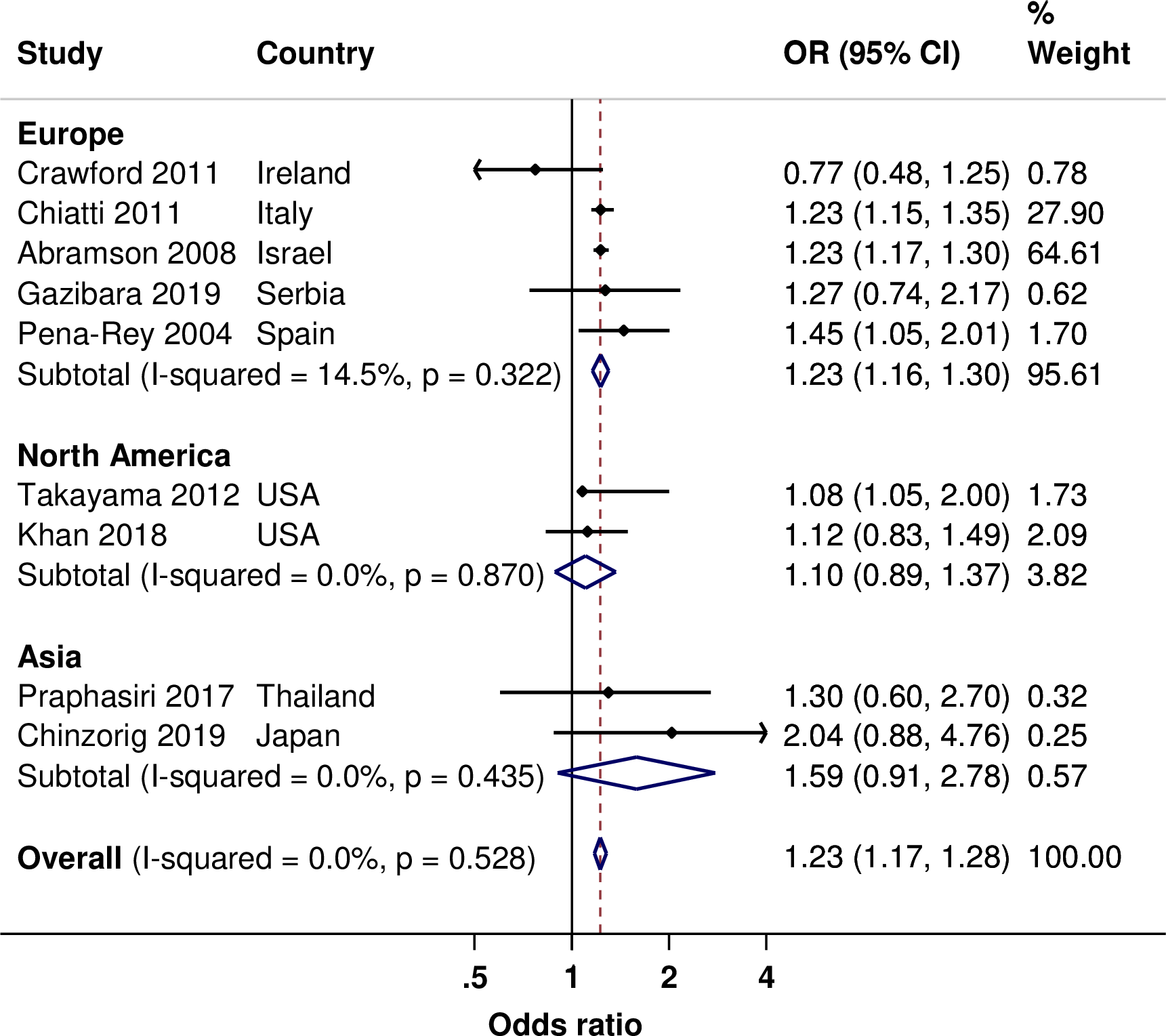

Supplement: S2 Fig — (TIF) [file pone.0234702.s003.tif]

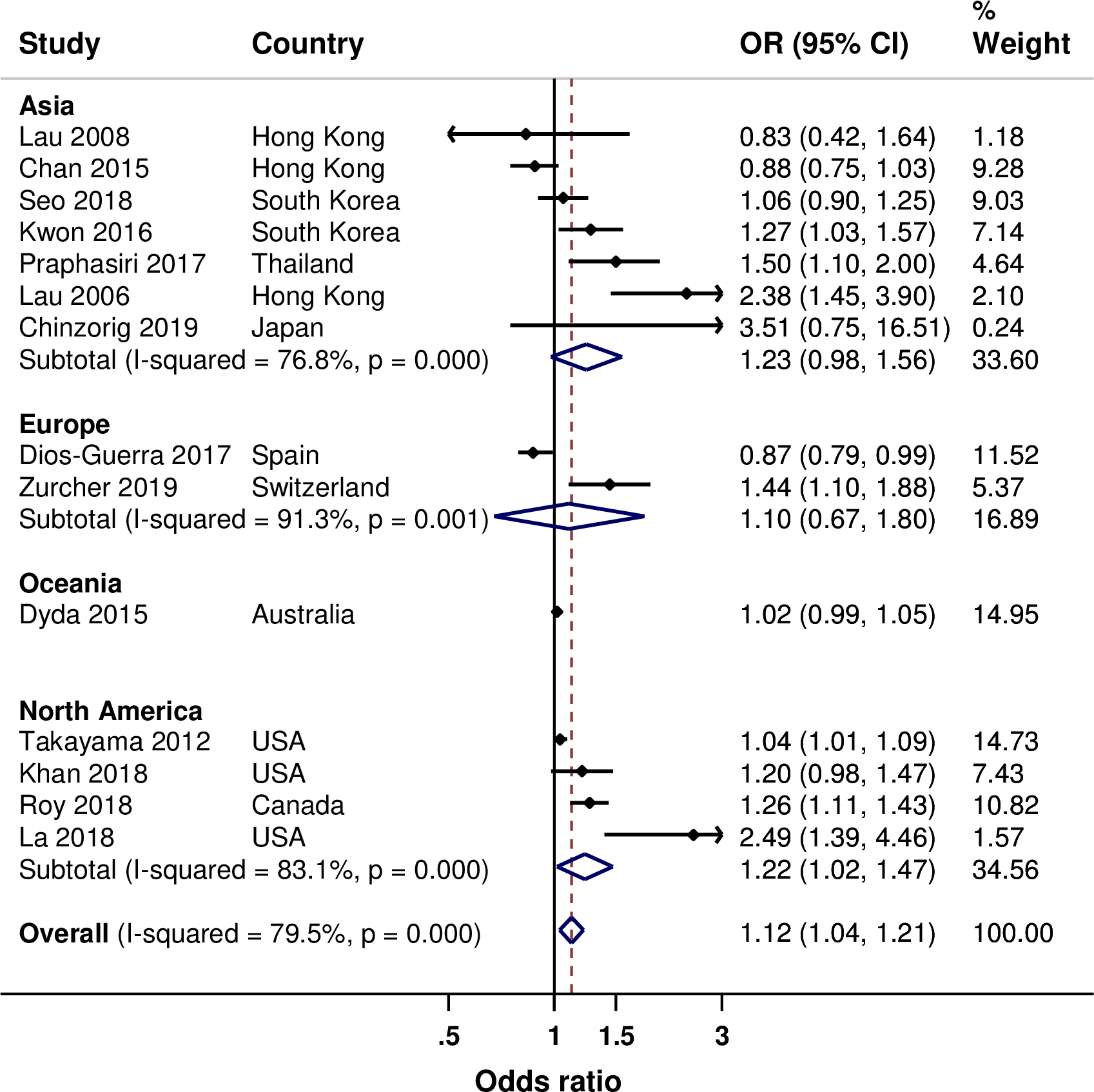

Supplement: S3 Fig — (TIF) [file pone.0234702.s004.tif]

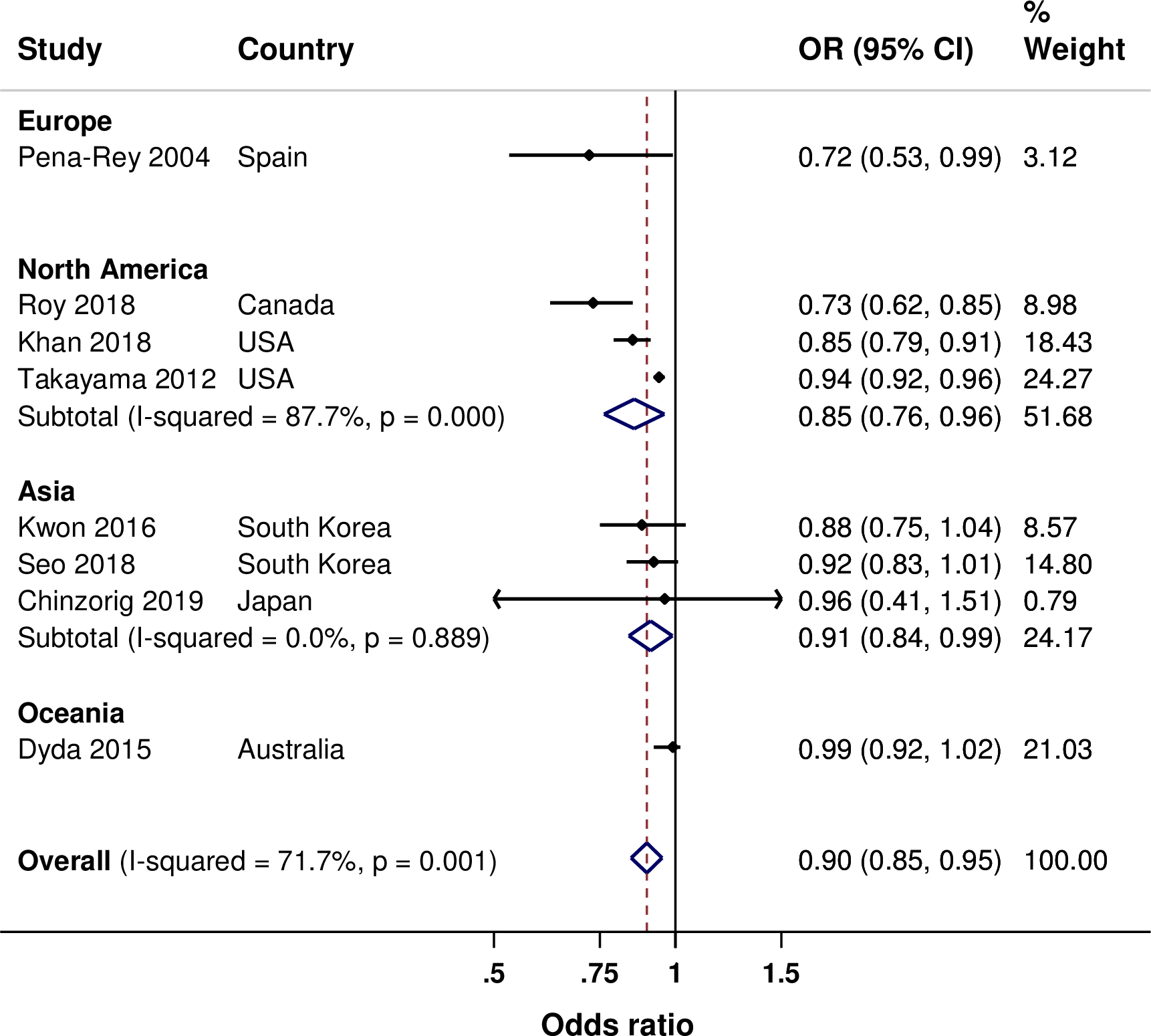

Supplement: S4 Fig — (TIF) [file pone.0234702.s005.tif]

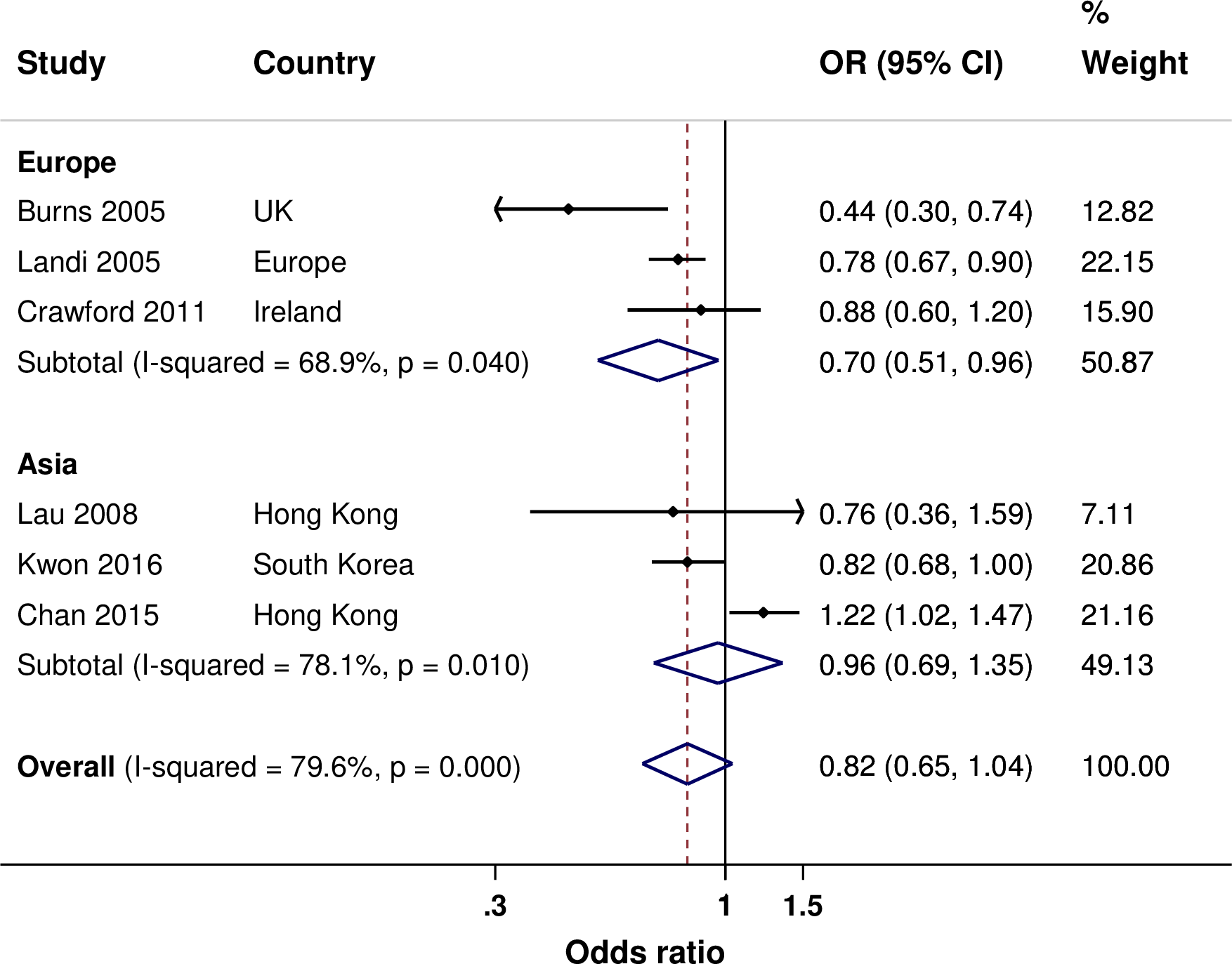

Supplement: S5 Fig — (TIF) [file pone.0234702.s006.tif]

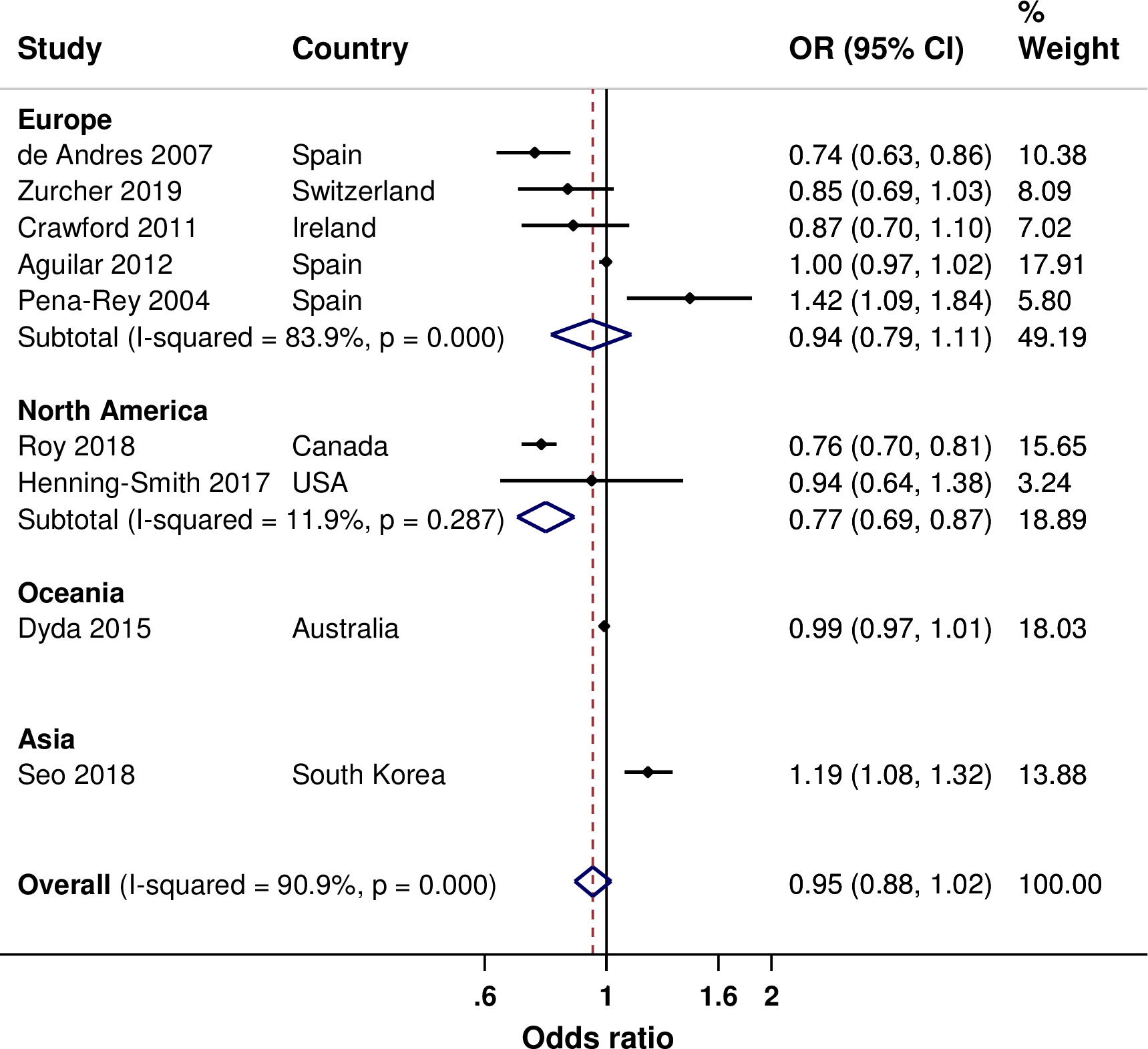

Supplement: S6 Fig — (TIF) [file pone.0234702.s007.tif]

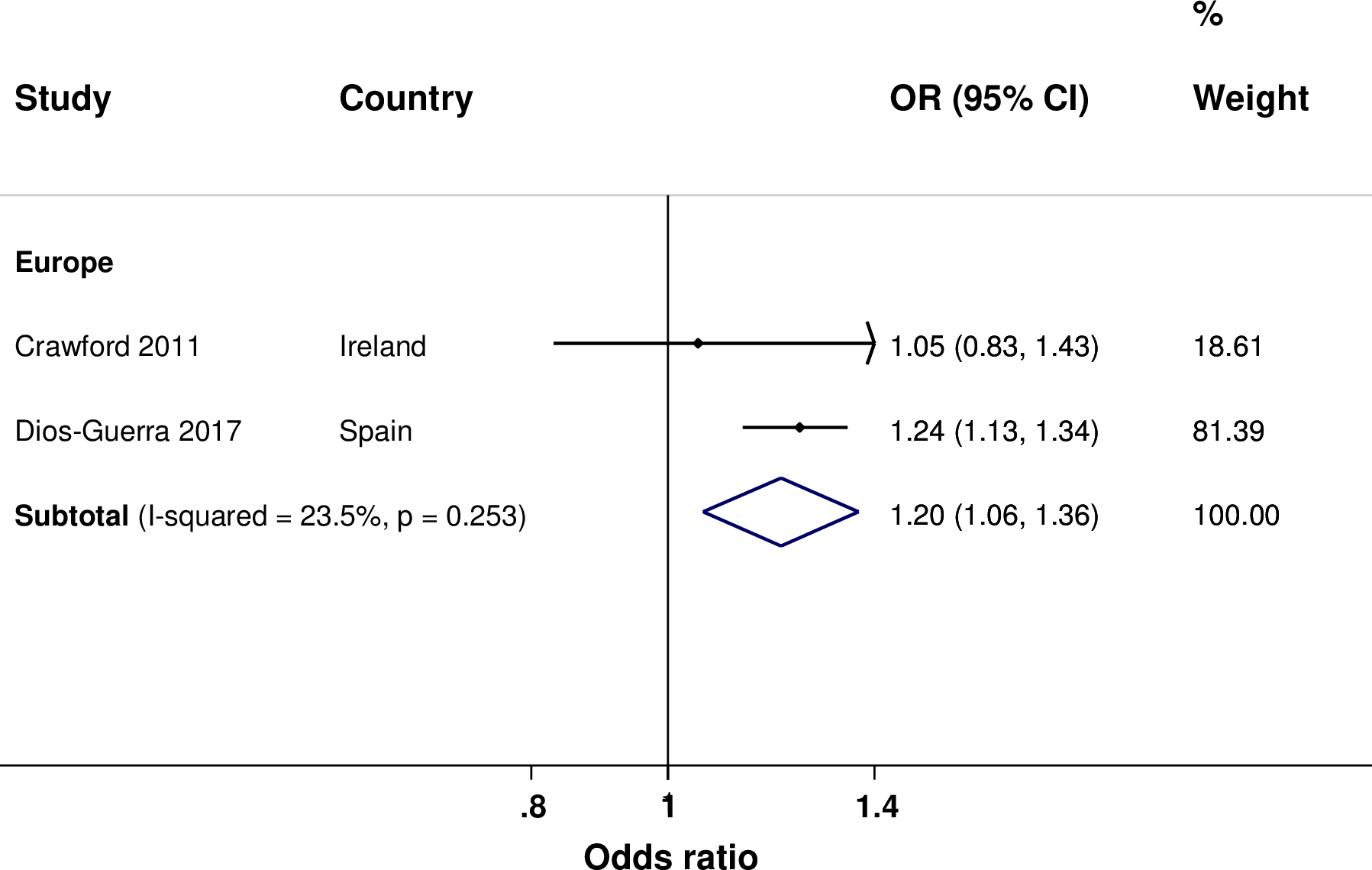

Supplement: S7 Fig — (TIF) [file pone.0234702.s008.tif]

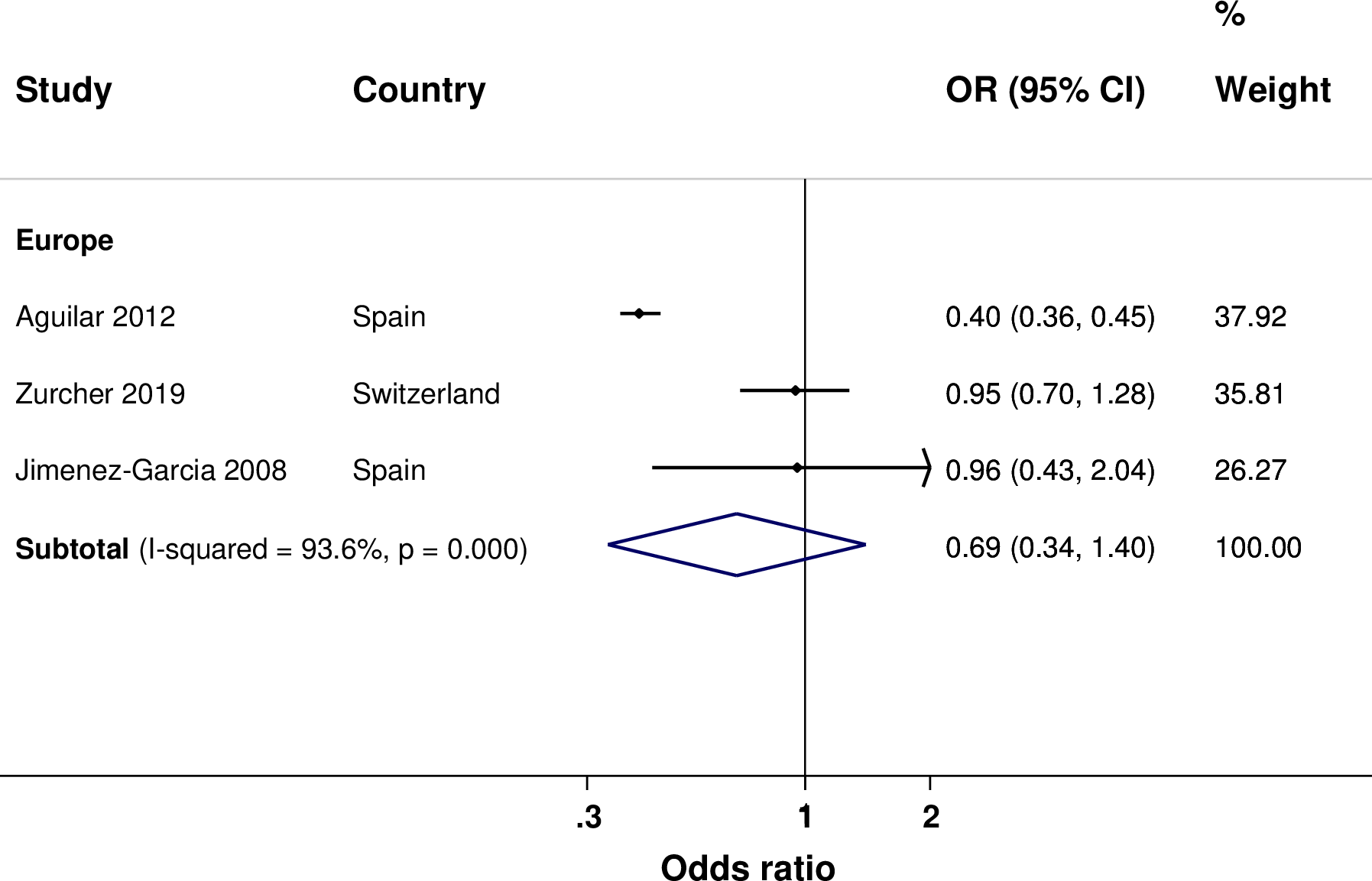

Supplement: S8 Fig — (TIF) [file pone.0234702.s009.tif]

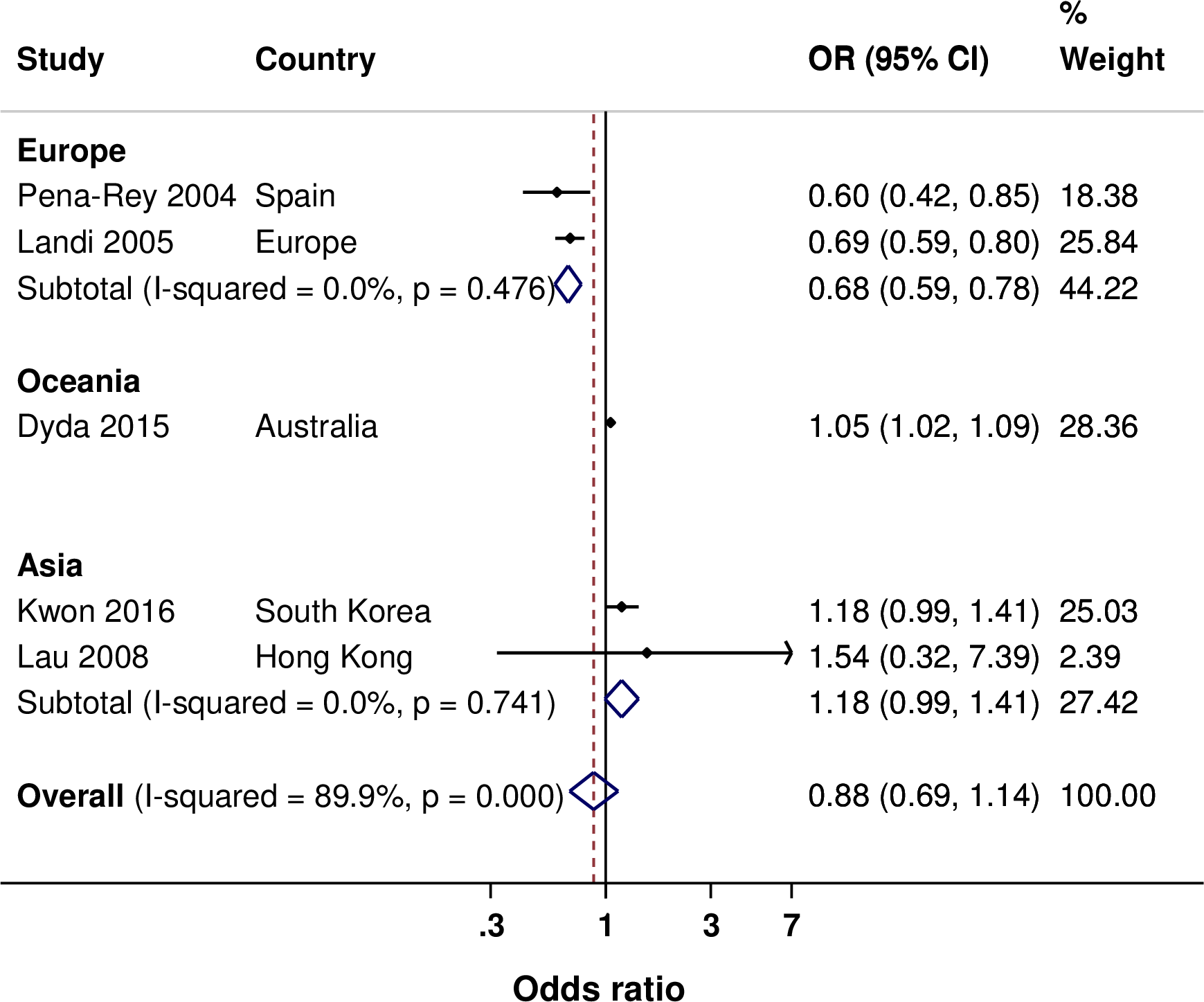

Supplement: S9 Fig — (TIF) [file pone.0234702.s010.tif]

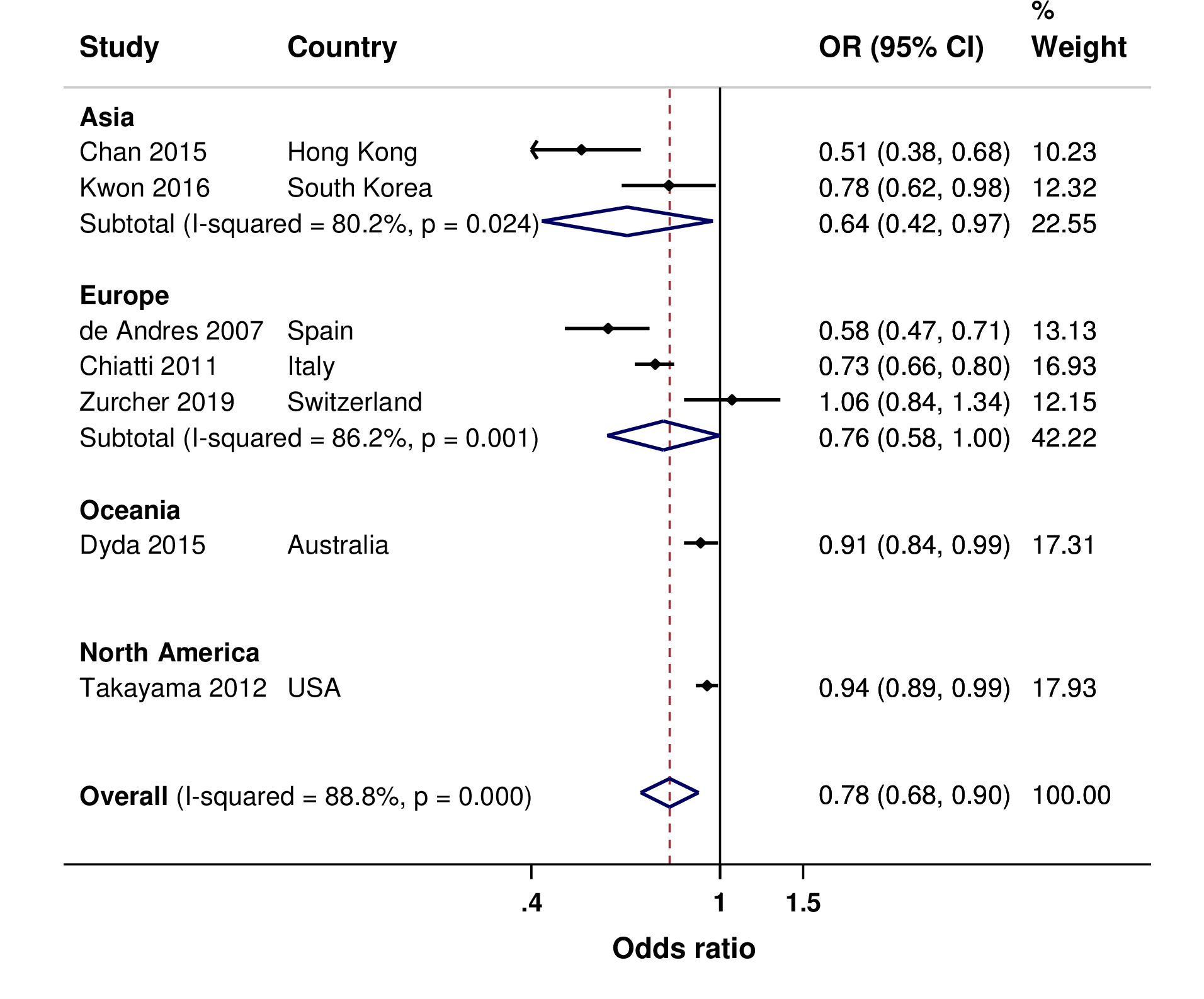

Supplement: S10 Fig — (TIF) [file pone.0234702.s011.tif]

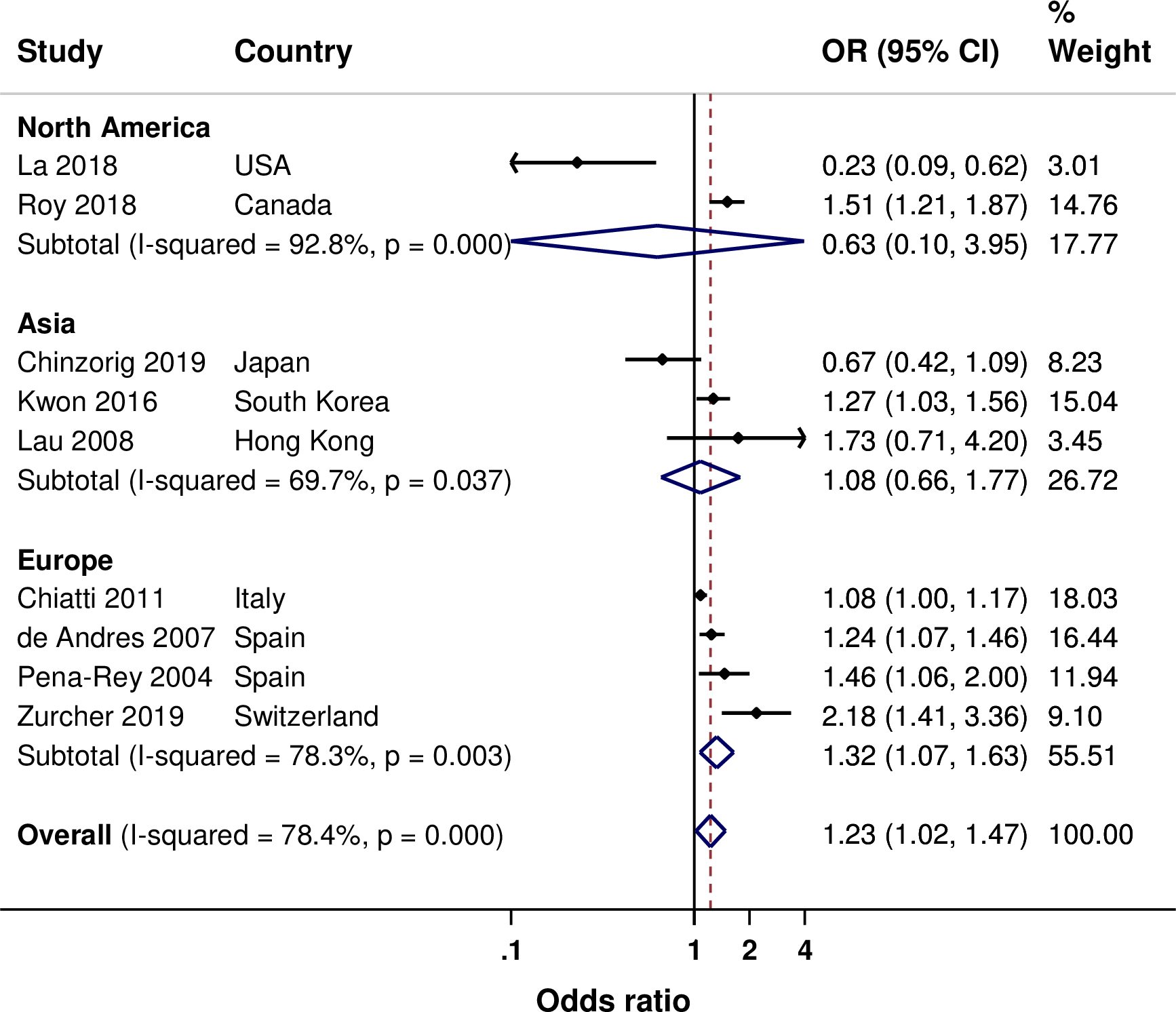

Supplement: S11 Fig — (TIF) [file pone.0234702.s012.tif]

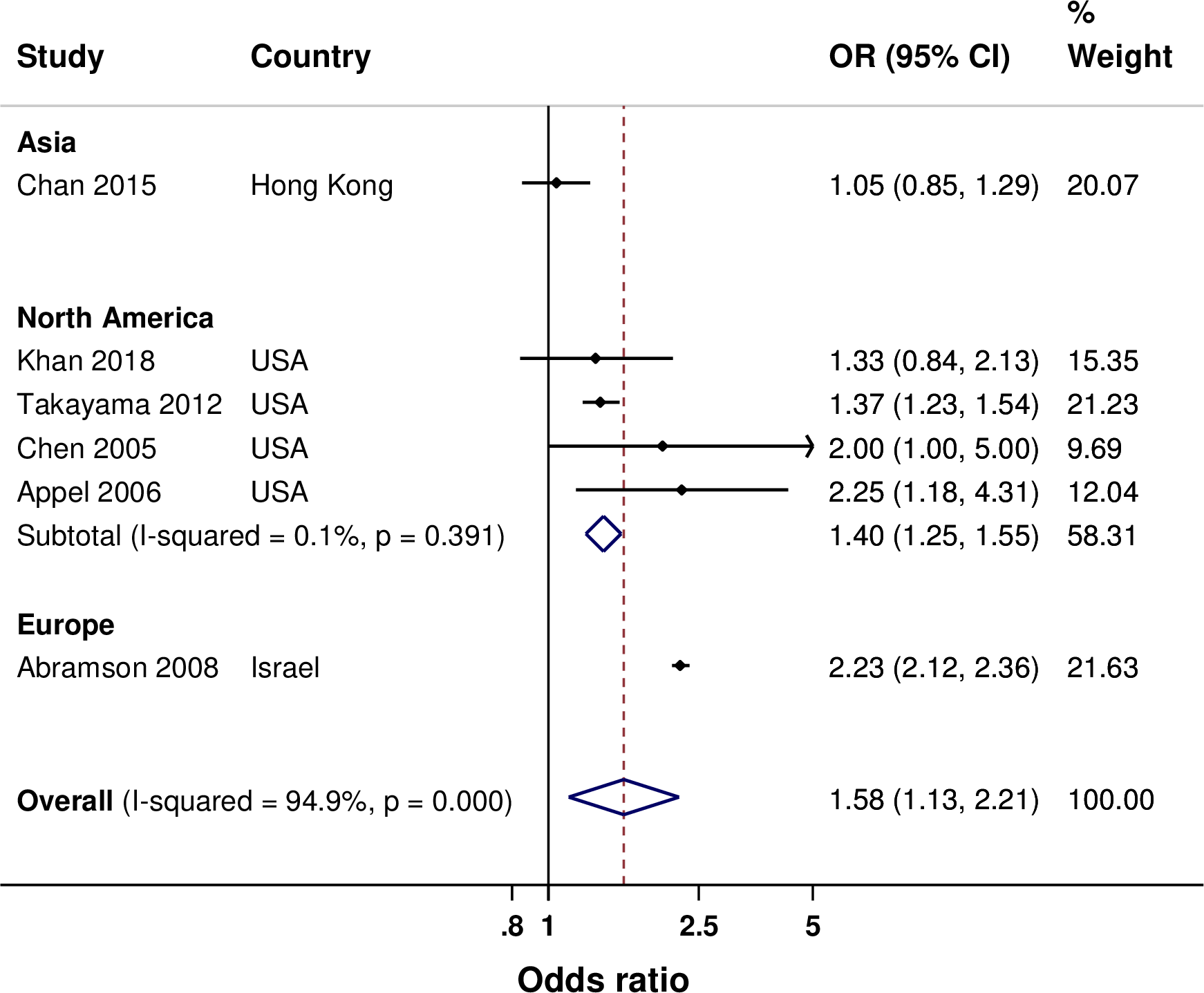

Supplement: S12 Fig — (TIF) [file pone.0234702.s013.tif]

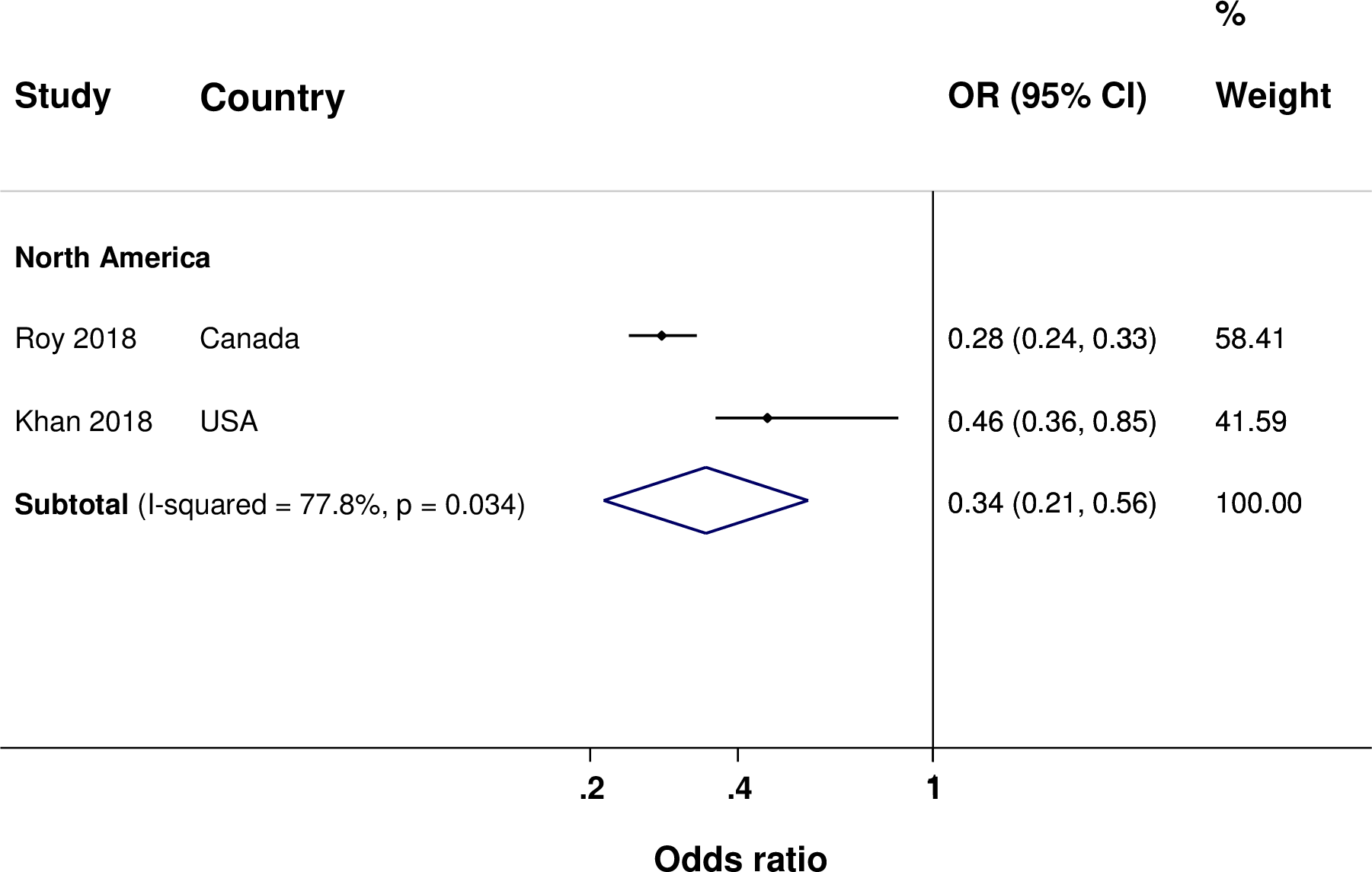

Supplement: S13 Fig — (TIF) [file pone.0234702.s014.tif]
